# Antioxidant Behavioural Phenotype in the *Immp2l* Gene Knock-Out Mouse

**DOI:** 10.3390/genes14091717

**Published:** 2023-08-28

**Authors:** Adam J. Lawther, Jerzy Zieba, Zhiming Fang, Teri M. Furlong, Illya Conn, Hemna Govindaraju, Laura L. Y. Choong, Nigel Turner, Khawar Sohail Siddiqui, Wallace Bridge, Sam Merlin, Tzipi Cohen Hyams, Murray Killingsworth, Valsamma Eapen, Raymond A. Clarke, Adam K. Walker

**Affiliations:** 1 Laboratory of ImmunoPsychiatry, Neuroscience Research Australia, Randwick, NSW 2031, Australia; 2 Department of Psychology, University of Rzeszow, 35-310 Rzeszow, Poland; 3 Discipline of Psychiatry and Mental Health, University of New South Wales, Sydney, NSW 2052, Australia; 4 Ingham Institute for Applied Medical Research, Sydney, NSW 2170, Australia; tzipi.cohen-hyams@inghaminstitute.org.au (T.C.H.);; 5 School of Biomedical Sciences, University of New South Wales, Sydney, NSW 2052, Australia; 6 Schizophrenia Research Laboratory, Neuroscience Research Australia, Randwick, NSW 2031, Australia; 7 Department of Pharmacology, School of Biomedical Sciences, University of New South Wales, Sydney, NSW 2052, Australia; 8 Victor Chang Cardiac Research Institute, Darlinghurst, NSW 2010, Australia; 9 School of Biotechnology and Biomolecular Sciences, University of New South Wales, Sydney, NSW 2052, Australia; 10 Medical Science, School of Science, Western Sydney University, Campbelltown, Sydney, NSW 2751, Australia; 11 NSW Health Pathology, Liverpool Hospital Campus, 1 Campbell Street, Liverpool, NSW 2107, Australia; 12 Academic Unit of Infant Child and Adolescent Services (AUCS), South Western Sydney Local Health District, Liverpool, NSW 2170, Australia; 13 Monash Institute of Pharmaceutical Sciences, Monash University, Parkville, VIC 3052, Australia

**Keywords:** IMMP2L, MitoQ, antioxidant, behaviour, Autism, Tourette’s syndrome, dopamine, amphetamine, oxidative stress, locomotion, GPD2, AlphaFold, Immp1l, heterodimer

## Abstract

Mitochondrial dysfunction is strongly associated with autism spectrum disorder (ASD) and the *Inner mitochondrial membrane protein 2-like (IMMP2L)* gene is linked to autism inheritance. However, the biological basis of this linkage is unknown notwithstanding independent reports of oxidative stress in association with both IMMP2L and ASD. To better understand *IMMP2L’s* association with behaviour, we developed the *Immp2l^KD^* knockout (KO) mouse model which is devoid of Immp2l peptidase activity. *Immp2l^KD^* −/− KO mice do not display any of the core behavioural symptoms of ASD, albeit homozygous *Immp2l^KD^* −/− KO mice do display increased auditory stimulus-driven instrumental behaviour and increased amphetamine-induced locomotion. Due to reports of increased ROS and oxidative stress phenotypes in an earlier truncated *Immp2l* mouse model resulting from an intragenic deletion within *Immp2l*, we tested whether high doses of the synthetic mitochondrial targeted antioxidant (MitoQ) could reverse or moderate the behavioural changes in *Immp2l^KD^* −/− KO mice. To our surprise, we observed that ROS levels were not increased but significantly lowered in our new *Immp2l^KD^* −/− KO mice and that these mice had no oxidative stress-associated phenotypes and were fully fertile with no age-related ataxia or neurodegeneration as ascertained using electron microscopy. Furthermore, the antioxidant MitoQ had no effect on the increased amphetamine-induced locomotion of these mice. Together, these findings indicate that the behavioural changes in *Immp2l^KD^* −/− KO mice are associated with an antioxidant-like phenotype with lowered and not increased levels of ROS and no oxidative stress-related phenotypes. This suggested that treatments with antioxidants are unlikely to be effective in treating behaviours directly resulting from the loss of Immp2l/IMMP2L activity, while any behavioural deficits that maybe associated with IMMP2L intragenic deletion-associated truncations have yet to be determined.

## 1. Introduction

Oxidative stress has been associated with a range of neurodevelopmental disorders and cognitive abnormalities including autism spectrum disorder (ASD), Gilles de la Tourette syndrome (GTS) and attention deficit hyperactivity disorder (ADHD) [1,2,3,4,5,6], however, the role that oxidative stress plays in these disorders is uncertain. Each of these neurodevelopmental disorders present with strong male gender bias and overlaps in their behavioural profiles and risk genes [7,8]. One such risk gene is *IMMP2L* which is located at the 7q31 chromosome locus that has been repeatedly linked with autism inheritance (Table 1) [9,10,11,12,13,14,15,16,17,18,19,20,21,22,23,24,25]. The precise biological basis of this linkage with ASD has yet to be determined [12]. 

A most interesting feature related to the many previous investigations of the earlier truncated *Immp2l*^Tg(Tyr)979Ove^ mouse model was that a number of its oxidative stress phenotypes were successfully reversed by administration of a synthetic mitochondrial targeted antioxidant named SkQ1 provided in drinking water [28,29]. To explore if oxidative stress is associated with the behavioural changes in our new *Immp2l^KD^* −/− KO mouse, we likewise administered a mitochondrial targeted antioxidant Mitoquinone (MitoQ) in the drinking water of mice from the time of weaning. MitoQ is a synthetically modified form of coenzyme Q_10_ bound with triphenylphosphonium-based cations that target MitoQ to the mitochondria making it more potent in lessening mitochondrial-derived oxidative damage than untargeted antioxidants. The SkQ1 used by Lu et al. [28,29] and the MitoQ used in this study show comparable efficacy in lowering oxidative stress when administered in drinking water [30,31]. Moreover, MitoQ is proven to be effective at lowering oxidative stress in mice [32,33,34] and in humans [30]. 

A ‘common chromosomal fragile site’ located within the *IMMP2L* gene [35] appears to contribute to the genes’ relative instability with high incidence of heterozygous deletions reported within *IMMP2L* in ASD and in normal ‘neurotypical’ populations (Table 2) [36]. A number of smaller studies had suggested that *IMMP2L* may be deleted at higher frequencies in ASD [13,37,38,39,40,41,42,43,44,45,46] (Table 2), Gillies de la Tourette syndrome (GTS) [40,47,48,49,50], attention deficit hyperactivity disorder, (ADHD) [44] and intellectual disability (ID) [45] populations (Table 3). Larger studies, however, indicate that the frequency of *IMMP2L* deletions (inclusive of exon sequence) in ‘neurotypical’ control populations is often higher (between 0.1% and 1.85%) [36] than those associated with ASD (between 0.27% and 1.54% [36]) and other neurodevelopmental disorders. Notwithstanding, autism is strongly associated with mitochondrial dysfunction [51,52] and *IMMP2L* encodes a key peptidase localized within the inner mitochondrial membrane that processes other mitochondrial proteins through the removal/cleavage of mitochondrial-specific signal peptides [3]. 

The nature and significance of any of *IMMP2L’s* behavioural associations remains uncertain and the effects of IMMP2L haploinsufficiency on human behaviour remains unknown. To this end, we developed the *Immp2l^KD^* −/− knockout (KO) mouse [53] to better evaluate the role of IMMP2L on behaviour and to determine if oxidative stress is a factor in the behaviours impacted by IMMP2L. In our earlier studies of behaviour we observed that *Immp2l^KD^* homozygous (−/−) and heterozygous (+/−) KO mice [53] do not exhibit any of the core behavioural symptoms of ASD with no evidence of impaired social interaction, repetitive behaviours, restricted interests or behavioural rigidity [54]. Albeit *Immp2l^KD^* −/− KO mice do display increased auditory stimulus-driven instrumental behaviour [55] and enhanced drug-induced locomotion in response to dexamphetamine that is both gene-dose dependent and sex-specific [54].

An earlier truncated *Immp2l* mouse model (*Immp2l*^Tg(Tyr)979Ove^) first reported in 2008 by Lu et al. [3] and which subsequently became the subject of at least 18 additional publications [3,28,29,56,57,58,59,60,61,62,63,64,65,66,67,68,69,70,71] presented with increased ROS and numerous associated oxidative stress phenotypes. Furthermore, this earlier truncated *Immp2l*^Tg(Tyr)979Ove^ mouse had a complex genotype (*Immp2l*^Tg(Tyr)979Ove^) inclusive of an intragenic deletion in *Immp2l* and expressed a largely intact yet truncated form of Immp2l that made it difficult to determine the precise origin of the increased levels of ROS and associated oxidative stress phenotypes [3]. With the emergence of our more recent *Immp2l^KD^* −/− KO mouse, which presents with no oxidative stress phenotypes, the genetic origin of the oxidative stress in the earlier truncated *Immp2l*^Tg(Tyr)979Ove^ mouse model is less certain. These oxidative stress phenotypes of the truncated *Immp2l*^Tg(Tyr)979Ove^ −/− mouse model included infertility defects affecting folliculogenesis and ovulation in females and defects in both spermatogenesis and erectile dysfunction in males, age-related neurodegeneration and ataxia—all of which are absent from our more recent *Immp2l^KD^* −/− KO mice. In addition, the earlier truncated *Immp2l*^Tg(Tyr)979Ove^ mouse was developed on a blind background strain of mice (FVB/N) that made it unsuitable for behavioural examination [3]. As our primary interest is the aetiology of ASD-relevant behaviours, we developed the new *Immp2l^KD^* −/− KO mouse strain on a C57BL/6J background [53] that we demonstrate is devoid of Immp2l peptidase activity yet exhibits none of the overt oxidative stress-related phenotypes reported for the earlier truncated *Immp2l*^Tg(Tyr)979Ove^ mouse model [3,28,29,56,57,58,59,60,61,62,63,64,65,66,67,68,69,70,71].

In the present study, we confirm increased locomotion in response to dexamphetamine in the *Immp2l^KD^* −/− KO mouse [54], and find no evidence of oxidative stress in *Immp2l^KD^* −/− KO mice or efficacy of the mitochondrial targeted antioxidant MitoQ in remediating the enhanced drug-induced locomotion of these mice. Furthermore, given that the phenotype of the *Immp2l^KD^* −/− KO mouse was in such dramatic contrast with that of the earlier truncated *Immp2l*^Tg(Tyr)979Ove^ mouse model reported by Lu et al. [3] we went in search of answers using the AlphaFold2-Multimer high-accuracy tool for predicting the quaternary structure of protein complexes. This analysis predicted with high confidence that Immp1l forms a heterodimer with the C-terminal truncated form of Immp2l expressed in the earlier *Immp2l*^Tg(Tyr)979Ove^ mouse model thereby providing a pathway for gain-of-function oxidative stress effects from within the inner mitochondrial membrane.

## 2. Materials and Methods

### 2.1. Animals and Ethics

Homozygous *Immp2l^KD^* −/− KO mice were generated on a C57BL/6J background strain as described by Fang et al. [53]. Heterozygous male and female *Immp2l^KD^* −/− KO mice were bred to produce the 120 homozygous (*Immp2l^KD^* −/− KO) and wild-type (*Immp2l*^+/+^) male mice used in this study (n = 30/group). Male mice were chosen for this study since the male *Immp2l^KD^* −/− KO mice have demonstrated the most profound locomotor changes in response to dexamphetamine challenge [54]. Mice were bred and group housed at the Ingham Institute Biological Resource Unit (Liverpool, NSW, Australia) in individually ventilated cages (GM500 Green, Techniplast Australia Pty Ltd., Rydalmere, NSW, Australia) with corn cob bedding, crinkle cut cardboard nesting material and a red igloo (Bioserv, Frenchtown, NJ, USA). At 4 months of age, mice were transported to either Neuroscience Research Australia (Randwick, NSW, Australia) for behavioural studies or to the University of New South Wales, (Sydney, NSW, Australia) for tissue and mitochondrial analyses. Mice were allowed to acclimate to the new facility for at least one month before testing and were housed in standard shoebox cages in a temperature and humidity-controlled environment with a 12/12 h modified dark-light cycle (lights on at 0700) with identical cage mates. Mice were housed in homogenous pairs to avoid fighting. Food and water or MitoQ-treated water were available ad libitum. Mice were randomly allocated to experimental groups. Mice were euthanized with CO_2_ or cervical dislocation. Tissues were dissected after perfusion with sterile PBS. All procedures involving mice were carried out under protocols approved by the UNSW Animal Ethics Committee (protocol numbers 19/6B, 15/48B and 18/78A) and in accordance with National Health and Medical Research Council guidelines. Animals were monitored daily. No unexplained mortality occurred in these studies. 

### 2.2. SDS–PAGE and Western Analysis

We used western analysis to investigate the cleavage of Immp2l substrates Cyc1 and Gpd2. Mouse tissues and mitochondria purified from these tissues (see section on *Mitochondrial isolation*) were freshly prepared in RIPA Buffer (Thermo Fisher Scientific, Rockford, IL, USA) containing a phosphatase inhibitor cocktail (Halt #78429 Thermofisher Scientific 1:100) before adding 1:1 2× Laemmli buffer (2% SDS, 10% glycerol, 5% 2-mercaptoethanol, 0.002% bromophenol blue, 0.0625 M Tris HCl). Protein samples (30 µg) were heated at 95 °C for 5 min before electrophoresis on precast TGX SDS-PAGE gels (Bio-Rad, Hercules, CA, USA) followed by Western transfer to 0.2 µM PDF membranes according to established protocols [72]. Membranes were probed with rabbit polyclonal antibodies against mouse proteins: Cyc1 (#10242-1-AP ProteinTech at 1:1000), Gpd2 (#17219-1-AP Proteintech at 1:500), Aifm (#VPA000017KT from Bio-Rad at 1:1000), Smac (#10434-1-AP Proteintech at 1:500), β-Actin (#20536-1-AP Proteintech at 1:3500) and Total OXPHOS Rodent WB antibody cocktail (#ab110413 Abcam at 1:250). Goat anti-rabbit horseradish peroxidase-conjugated secondary (#SA00001-2 Proteintech at 1:2000) was used with Luminol chemiluminescent reagent (Bio-Rad) to visualize proteins using the LI-COR model 2800 imaging system.

### 2.3. Antioxidant Treatment

Mitoquinone (MitoQ) is an orally active antioxidant with the ability to target free radicals within mitochondria reducing oxidative stress levels in mitochondria and the cytosol. MitoQ is currently under development by Antipodean Pharmaceuticals Inc. (Patent US-06331532 18 December 2001) and has been in phase II clinical trials for Parkinson’s disease and liver damage associated with HCV infection. MitoQ has demonstrated encouraging preclinical results in isolated mitochondria, cells and tissues undergoing oxidative stress and apoptotic death, preclinical trials in mice and in human clinical trials [30]. MitoQ was initially designed to mimic the role of the endogenous coenzyme Q10 (CoQ or Q_10_) in the electron transport chain within the inner mitochondrial membrane [30]. While it is doubtful that MitoQ functions as an effective electron carrier within the electron transport chain, it has been proven to be effective as a free radical scavenger within mitochondria, effectively augmenting the antioxidant capacity of the endogenous coenzyme Q10 to supraphysiological levels [30,33,34]. MitoQ was prepared in sterilised filtered water at a working concentration of 0.20 mg/mL (~0.29 mM) and delivered in drinking water from the day of weaning until the end of experimental procedures at 6–7 months of age. The dose of MitoQ was titrated incrementally to the maximum dose in four increments (0.05, 0.10, 0.15 and 0.20 mg/mL each week) over the first 4 weeks after weaning. Fresh MitoQ solution or water were replenished twice weekly.

### 2.4. Behavioural Studies

Behavioural tests were conducted to examine anxiety-like behaviour (open field and elevated-plus maze), fear conditioning, sensorimotor gating (pre-pulse inhibition; PPI), sensitivity to amphetamine-induced hyperlocomotion, and grooming behaviour.

#### 2.4.1. Elevated Plus-Maze

Anxiety-like behaviour was assessed using the elevated plus maze (EPM), which comprised of two enclosed arms with walls (length x width x height: 35 × 6 × 28 cm) and two open arms without walls with the same length and width dimensions. The central platform was a 6 × 6 cm square. The EPM was raised 70 cm above the floor. Mice were placed onto the central platform facing an enclosed arm and allowed to freely explore for 5 min. The maze was dimly illuminated at 10 lux. Behaviour was recorded by video camera, and distance travelled, arm entries and time spent in each arm was assessed using ANY-maze Video Tracking System Version 4.99z (Stoelting Co., Wood Dale, IL, USA). An arm entry was recorded when at least half the body length of the mouse entered an arm.

#### 2.4.2. Open Field Test

Anxiety-like behaviour was assessed in square activity chambers (43 × 43 cm) from Med Associates Inc. (St Albans, VT, USA). Mice were placed in the corner of the chamber and allowed to freely explore for 30 min. Infrared photobeams were used to assess activity. Horizontal (distance travelled) and vertical activity (rearing) in central and peripheral zones were assessed. The ratio of central to total distance travelled (distance ratio) and time spent in the central area of the open field were taken as measures of anxiety. Photobeam breaks when mice were not ambulating or rearing (i.e., did not meet the criteria for either horizontal or vertical activity) was interpreted as an indication of non-ambulatory movement (referred to as ‘stereotypic behaviour’ in the MedAssociates Inc. software Version 6.02, St Albans, VT, USA).

#### 2.4.3. Dexamphetamine-Induced Hyperlocomotion

Dexamphetamine increases locomotion and rearing in some ASD mouse models [73]. Mice were placed into the square activity chambers (as described above) and allowed to freely explore the arena for 30 min, at which point they were removed briefly and injected with dexamphetamine (2 mg/kg, IP; National Measurement Institute, Australia) dissolved in sterile PBS. After injection, mice were immediately returned to the activity chamber and allowed to explore for a further 60 min. Horizontal and vertical activity, and stereotypic behaviour was assessed identical to that described above for the open field test.

#### 2.4.4. Fear Conditioning

Cued and contextual fear conditioning were assessed. On the first day, mice were placed in the test chamber (Model H10-11R-TC, Coulbourn Instruments, Whitehall, OH, USA) for 120 s before an 80 decibel (dB) tone (conditioning stimulus) was presented for 30 s. A co-terminating 0.4 mA 2 s foot shock (unconditioned stimulus) occurred twice with an inter-pairing interval of 120 s and the test concluded 120 s later. To assess contextual fear-conditioned memory, mice were returned to the apparatus for 7 min on the second day. Cued fear conditioned memory was assessed on the third day by placing mice in a new chamber distinct from the previous two days for 9 min. After 120 s, the tone conditioning stimulus was continuously presented for 5 min, and the test concluded 120 s after the termination of the tone. White noise (68 dB) was used as background noise. Time spent freezing was assessed using ANY-maze Video Tracking System Version 4.99z (Stoelting Co., Wood Dale, IL, USA).

#### 2.4.5. Pre-Pulse Inhibition (PPI)

Sensorimotor gating, as indicated by the attenuation of the startle response by a non-startling stimulus, was measured using SR-LAB startle chambers (San Diego Instruments, San Diego, CA, USA). The PPI test consisted of exposure to 70 dB background noise for 5 min before 97 of the following trials were presented in a pseudorandom order: 5 × 70 dB trials (background); 5 × 100 dB trials; 15 × 120 dB trials [startle; separated into blocks of 5 startle trials each (first, middle and last startle block)] and 6 x trials, which included a prepulse of either 74, 82 or 86 dB presented either 32, 64, 128 or 254 ms (variable interstimulus interval (ISI)) prior to a startling pulse of 120 dB (to elicit the PPI response). The interval between trials differed randomly between 10 and 20 s. Acoustic startle responses were calculated as mean amplitude to all startle trials. Percentage of PPI (%PPI) was calculated as mean startle response (120 dB)-PPI response/mean startle response (120 dB)] × 100 and averaged across ISIs to produce mean percentage PPI for each prepulse intensity. 

#### 2.4.6. Grooming Behaviour

Mouse grooming behaviour typically follows a predefined four-phase pattern known as a syntactic chain. This pattern starts with short elliptical strokes to the nose (phase 1), unilateral strokes to the face (phase 2), large bilateral strokes over the ears (phase 3), and ending with body/flank grooming (phase 4) [74]. The assessment of grooming behaviour is a valuable tool for translational neuroscience (reviewed in [75]), and changes in grooming have been observed in preclinical models of ASD [76], and Tourette’s syndrome [77]. To determine if the stereotypic differences observed in the open field were a result of changes in the amount of grooming or in grooming pattern rigidity, we assessed mice for the amount of time spent grooming, number of grooming bouts, and sequential stereotypy (adherence to the syntactic chain) using the grooming splash test. Mice were placed into glass beakers (8.5 cm diameter × 12 cm high) and recorded on digital video cameras positioned directly in front of the beakers. Mice habituated to the environment for 10 min before being misted with room temperature water from a spray bottle to encourage grooming [77], and recorded for a further 10 min. To assess the effects of amphetamine administration on grooming behaviour, mice were misted with water following dexamphetamine and recorded for 10 min after habituation to the beaker. An experimenter blind to treatment scored the videos in real-time and recorded total grooming time and completed grooming bouts. A grooming sequence was considered complete and used as a proxy for sequential stereotypy when face grooming transitioned to flank grooming, and incomplete when grooming ended without flank grooming or face grooming.

### 2.5. Assessment of MitoQ Brain and Kidney Concentration

The assessment of brain and kidney MitoQ concentrations was conducted by the Mitochondrial Dysfunction Laboratory, Cambridge, UK. Samples were shipped on dry ice from Australia to the UK. Each sample was spiked with 10 µL of internal standard (10 µM d_15_-MitoQ). Standards were spiked additionally with 10 µL of the MitoQ stocks of varying concentration to achieve a standard curve. LC-MS analysis was performed using Waters Xevo TQ-S mass spectrometer (Waters, Wilmslow, UK), samples were stored in a cooled autosampler and injected at 2 µL. Separation was achieved using an Acquity UPLC BEH 1.7µM C18 Column (Waters, UK). A flow rate of 0.200 µL/min was used and mobile phases of A) 95% H_2_O/5% Acetonitrile/0.1% Formic Acid and B) 90% Acetonitrile/10% H_2_O/0.1% Formic Acid. Spectra data was processed using MassLynx. All data were calculated based on MS response relevant to the internal standards. 

### 2.6. Assessment of Mitochondrial Reactive Oxygen Species

#### 2.6.1. Mitochondrial Isolation

The isolation of mitochondria followed methods previously described [78,79,80]. Immediately after excision, kidney and brain tissue were prepared by finely dicing in ice-cold isolation buffer A (250 mM sucrose, 10 mM Tris-HCl, 1 mM EGTA, 1% fatty-acid free BSA, pH 7.4). The tissue was rinsed with buffer A, before homogenisation using a glass-Teflon Dounce homogeniser. Heart tissue was similarly prepared in ice-cold isolation buffer B (100 mM sucrose, 100 mM KCl, 50 mM Tris-HCl, 1 mM KH_2_PO_4_, 1 mM EGTA, 0.2% fatty-acid free BSA, pH 7.0). The heart tissue was incubated for 2 min in 1 mg/mL of proteinase, and rapidly rinsed in buffer A to stop digestion. The heart tissue was then homogenised using a Polytron homogeniser (Thomas Scientific, Swedesboro, NJ, USA). Homogenates were centrifuged at 1000× *g* for 5 min at 4 °C. The supernatant was collected and centrifuged at 10,000× *g* for 10 min at 4 °C. Pellets were washed in isolation buffer and centrifuged again at 10,000× *g* for 10 min at 4 °C. The pelleted mitochondrial isolate was resuspended in BSA-free ice-cold isolation buffer A and protein quantified using the bicinchoninic acid (BCA) assay (Thermo Fisher Scientific, Rockford, IL, USA).

#### 2.6.2. Hydrogen Peroxide/Superoxide Production in Isolated Mitochondria

The production of hydrogen peroxide (H_2_O_2_) and superoxide from kidney, brain and heart isolated mitochondria was indirectly measured by the oxidation of Amplex UltraRed Reagent (Invitrogen, Mount Waverley, VIC, Australia), as previously described [80]. In brief, 50 µg of mitochondrial protein/well was incubated in assay medium (120 mM KCl, 3 mM HEPES, 1 mM EGTA, 0.3% fatty-acid free BSA, pH 7.2) warmed to 37 °C. Shortly before the assay, the assay reagent (assay medium supplemented with 50 µM Amplex UltraRed Reagent, 120 U/mL superoxide dismutase, 6 U/mL horseradish peroxidase and 100 µM phenylmethylsulfonyl fluoride) was added to each well. The kinetic assay was started with addition of substrate, succinate (5 mM), or succinate in the presence of rotenone (2 µM). The plate was read in a FlexStation 3 Multi-Mode Microplate Reader (BMG) with excitation/emission wavelengths of 544 nm/590 nm for 30 min at 37 °C. Each plate also contained wells with assay medium and reagent containing a range of H_2_O_2_ concentrations from 0 to 1600 pmol in order to generate a standard curve.

### 2.7. Electron Microscopy (EM) of the Adult Mouse Brain

#### 2.7.1. Tissue Processing for Electron Microscopy

Medial prefrontal cortex from adult mice 4–5 months of age were routinely processed as for TEM imaging, which included fixation with 2.5% glutaraldehyde in 0.1 M sodium cacodylate buffer pH 7.4. After fixation, the tissue was rinsed in sodium cacodylate buffer for 1 to 2 days and then processed routinely for electron microscopy. This included 4 h 2% osmium tetroxide, 1 h 2% uranyl acetate, 1 h sodium acetate, dehydration through graded alcohol and acetone followed by impregnation with Spurr low-viscosity epoxy resin standard formulation. The resin was then cured at 70 °C for 15 h [81].

#### 2.7.2. Field Emission Scanning Electron Microscopy

For sub-nanometer field emission scanning electron microscopy (FESEM), ~200 nm thick sections were cut with an ultramicrotome (RMC Boeckeler, Tucson, AZ, USA) fitted with an Ultra 45 diamond knife (Diatome, Nidau, Switzerland) and mounted on silicon wafer substrate. The samples were carbon coated using an automatic SEM carbon coater (Agar Scientific, London, UK). Acquisition of 2D large area electron microscope (EM) images of the resin sections were performed using Atlas 5.2 in tile scan mode attached to the GeminiSEM 300 (Carl Zeiss, Jena, Germany) equipped with field emission gun. The Atlas system was used to control automated and pre-defined image acquisition, tiling, image-stitching and shade correction. Images were obtained using the scanning transmission electron microscopy (STEM) detectors at 30 kV at a working distance of 2.8 mm with a scan resolution of 2 nm/pixel. The STEM image can easily be correlated with the image of conventional histology if heavy metal stains are used and if the image contrast is reversed in the final display. The ability of this technique to discriminate between elements of different atomic numbers has obvious potential in many types of study. All images have been inverted to give a TEM-like appearance. Pre-checked imaging conditions were optimised in order to maximise brightness and contrast and avoid astigmatism for each image [81]. 

### 2.8. AlphaFold2-Multimer High-Accuracy Prediction of Complex Structures

High-accuracy prediction of protein structures and associated oligomerization states were carried out using the AlphaFold2-Multimer program in ChimeraX (v 1.6.1) available at https://www.rbvi.ucsf.edu/chimerax, accessed 10 August 2023 [82] using default settings. Analysis of intermolecular contacts and visualization of structures were performed in ChimeraX [83] with predicted aligned error (PAE) plots interpreted as described [84]. 

### 2.9. Statistical Analysis

Mitochondrial respiration data were analysed using unpaired two-tailed *t*-tests to detect effects of genotype (*Immp2l^KD^* −/− KO vs. WT). Behavioural data were analysed using two-way univariate ANOVAs to detect effects of genotype (*Immp2l^KD^* −/− KO vs. WT) and treatment (MitoQ treated water vs. tap water). Where significant main or interaction effects were found, post-hoc Fisher’s LSD tests were used to reveal pairwise differences between groups. Amphetamine induced hyperlocomotion and PPI data were analysed using mixed design repeated measures ANOVAs with genotype and treatment as the between-subject variables and pre-pulse intensity (74, 82 and 86 db) or time as the within-subjects variable. A Greenhouse–Geisser correction epsilon (ε) was used to correct for potential violation of the sphericity assumption for the within-subjects’ measure. Data are presented as mean ± SEM. Statistical significance was set at *p* < 0.05 (two-tailed).

## 3. Results

### 3.1. Immp2l^KD^ −/− KO Mouse Model 

The generation of the *Immp2l^KD^* KO mouse strain was described previously together with the PCR genotyping strategy [53]. *Immp2l^KD^* heterozygotes and homozygotes appeared to develop normally beyond 6 months of age in comparable fashion with their wild-type littermates with no evidence of infertility (Table 4 and Table 5) with homozygous females and males breeding longer than expected, i.e., beyond 10 months of age and 17 months of age, respectively, with no evidence of ataxia or any other major health problems. The genotype ratios of offspring from mating 40 homozygote x homozygote mice and 62 heterozygote x heterozygote mice indicated normal fertility and Mendelian inheritance (Table 4 and Table 5). Previous behavioural testing of this *Immp2l^KD^* mouse line [54,55] identified behavioural differences compared with wild-type littermates with the strongest differences presenting in male *Immp2l^KD^* −/− KO mice [54].

### 3.2. IMMP2L Activity Absent from Immp2l^KD^ −/− KO Mice

**Figure 1:**
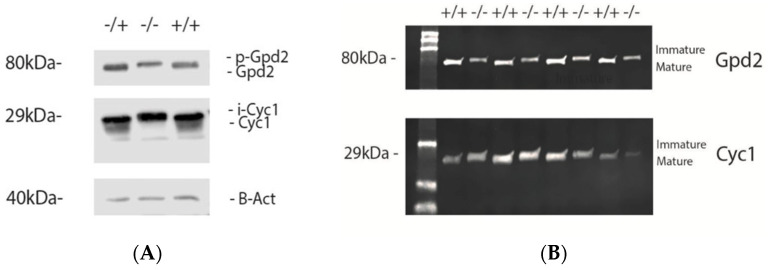
Western blot analysis of Immp2l substrates in brain and kidney of *Immp2l^KD^* −/− KO male mice compared with their heterozygous (+/−) and wild-type (+/+) littermates indicates no Immp2l peptidase activity in *Immp2l^KD^* −/− KO mice. (**A**) No cleavage of Immp2l substrates in kidney tissue of *Immp2l^KD^* −/− KO mice compared with *Immp2l^KD^*−/+ heterozygote and wild-type littermates that both display full cleavage of Cyc1 and Gpd2. (**B**) No Immp2l cleavage of Cyc1 of Gpd2 substrates in mitochondria purified from the brain of *Immp2l^KD^* −/− KO mice.

We used western analysis to evaluate the substrate specificity and activity of Immp2l in these mice. We found no evidence of Immp2l peptidase activity in the tissues or mitochondria from *Immp2l^KD^* −/− KO mice (Figure 1A,B). In contrast, Immp2l peptidase activity appeared normal in *Immp2l^KD^* −/+ heterozygotes in comparable fashion to their wild-type littermates (Figure 1A). To determine the substrate specificity and activity level of the Immp2l peptidase in *Immp2l^KD^* −/− KO mice, we used SDS PAGE and western analysis of proteins from brain and kidney and from mitochondria purified from these tissues, and compared the size and volume/density of the cleavage products/bands from IMMP2L reported substrates Cyc1, Gpd2 and Aifm [3,63,85] between *Immp2l^KD^* −/− KO male mice and their heterozygous and wild-type littermates (n = 4/group) (Figure 1A,B).

The Cyc1 protein is a component of the mitochondrial respiratory chain that is localised within the inner mitochondrial membrane (IMM). Cyc1 is the catalytic core subunit of the cytochrome c complex (Complex III) of the electron transport chain. Cyc1 is a nuclear-encoded protein synthesized in the cytosolic ribosome as a precursor protein (p-Cyc ~35kDa) with an N-terminal signal peptide that determines Cyc1’s localisation within the mitochondria of the cell. Cyc1 localises to the mitochondrial matrix before localisation within the IMM where it functions within the electron transport chain [3,63,85]. Cyc1 is cleaved in a two-step process [85]. The first cleavage of the Cyc1 precursor p-Cyc1 occurs in the matrix of the mitochondria by the MAS metallo-protease which gives rise to an intermediate form of Cyc1 (i-CYC1 ~ 29kDa). The subsequent cleavage of i-Cyc1 occurs in the IMM by Immp2L which generates the mature form of Cyc1 (m-Cyc1~26kDa). In our western analysis of proteins from brain and kidney, and from mitochondria purified from these tissues, we observed accumulation of only the immature intermediate form of Cyc1 (i-Cyc1 ~29kDa) in *Immp2l^KD^* −/− KO mice but not in their heterozygous or wild-type littermates (Figure 1A,B) which had only the mature form of Cyc1 (m-Cyc1 ~26kDa) (Figure 1A,B). From these observations, we concluded that *Immp2l^KD^* −/− KO mice do not process i-Cyc1 and were thus devoid of Immp2l peptidase activity.

Gpd2 functions as an integral component of the Glycerol Phosphate Shuttle (GPS) with its interactive cooperation between two closely related component molecules Gpd1 (cytosolic component) and its isozyme Gpd2 (mitochondrial component) with FAD as a cofactor. The GPS reduces quinone to quinol, recycles NADH to NAD+ for glycolysis and provides a supply of electrons for the electron transport chain through the production of FADH_2_ from the cyclical conversion of dihydroxyacetone phosphate (DHAP) (derived from glycolysis) to glycerol 3-phosphate (G3P) and back to DHAP. Gpd2 is a nuclear-encoded mitochondrial protein synthesized within the cytosolic ribosome as a precursor protein (p-Gpd2 ~81kDa) with a mitochondrial signal peptide sequence [3,63,85]. p-Gpd2 is translocated to the IMM where its signal peptide is cleaved-off in a single step proteolysis by Immp2l to yield the mature form of Gpd2 (m-Gpd2 ~76kDa). In our western analysis of proteins from brain and kidney, and from mitochondria purified from these tissues, we observed the accumulation of only the immature precursor form of Gpd2 (p-Gpd2 ~81kDa) in *Immp2l^KD^* −/− KO mice but not in their heterozygous or wild-type littermates (Figure 1A,B) that had only the fully processed (mature) form of Gpd2 (mGpd2 ~76kDa) in their mitochondria (Figure 1A,B). Consistent with our findings for Cyc1, these results confirmed that *Immp2l^KD^* −/− KO mice were devoid of Immp2l peptidase activity thus confirming their status as full Immp2l knockout (KO) mice and that any associated phenotypes are Immp2l KO phenotypes. No change in the size of Aifm was detected in the brain of either wild-type or *Immp2l^KD^* −/− KO mice.

### 3.3. Immp2l^KD^ −/− KO Enhances Dexamphetamine-Induced Hyperlocomotion

**Figure 2:**
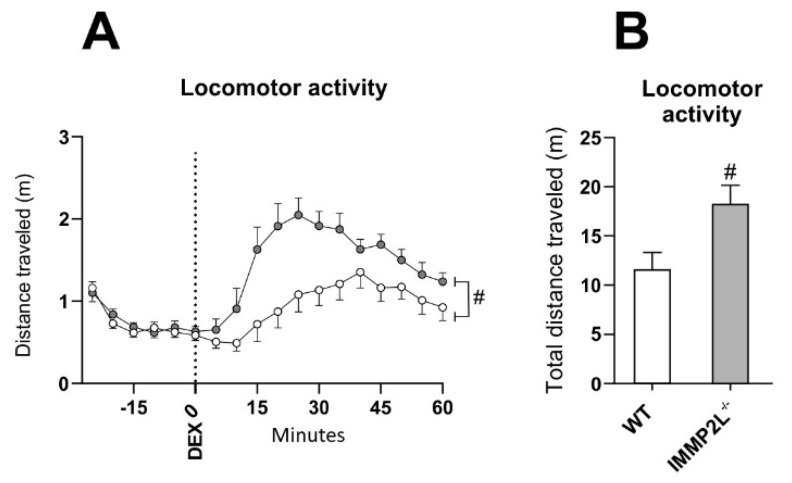
*Immp2l^KD^* −/− KO enhances dexamphetamine-induced locomotion. (**A**) Mean distance travelled throughout the dexamphetamine locomotor activity test before and after dexamphetamine for *Immp2l^KD^* −/− KO and wild-type mice. (**B**) Mean total distance travelled following dexamphetamine for *Immp2l* −/− and wild-type mice. # *p* < 0.05 for genotype differences. Error bars represent SEM.

To confirm *Immp2l^KD^* −/− KO mice show robust increases in dexamphetamine-induced locomotion [54], we assessed the impact of dexamphetamine on locomotor activity in *Immp2l^KD^* −/− KO and WT littermate mice exposed to untreated water. We replicated previous findings showing that while amphetamine increased locomotion in all mice (time main effect: *F*_(2.75,38.55)_ = 16.86, *p* < 0.001, ε = 0.25; Figure 2A), *Immp2l^KD^* −/− KO mice displayed significantly higher dexamphetamine-induced locomotion (time × genotype interaction: *F*_(2.75,38.55)_ = 3.69, *p* = 0.023, ε = 0.25; genotype main effect: *F*_(1,14)_ = 7.30, *p* = 0.017; Figure 2A,B) [54]. *Immp2l^KD^* −/− KO did not affect vertical activity (rearing) as previously described [54].

### 3.4. Antioxidant Treatment Using MitoQ Does Not Reverse the Behavioural Changes Associated with Immp2l^KD^ −/− KO on Dopamine-Mediated Behaviour

**Figure 3:**
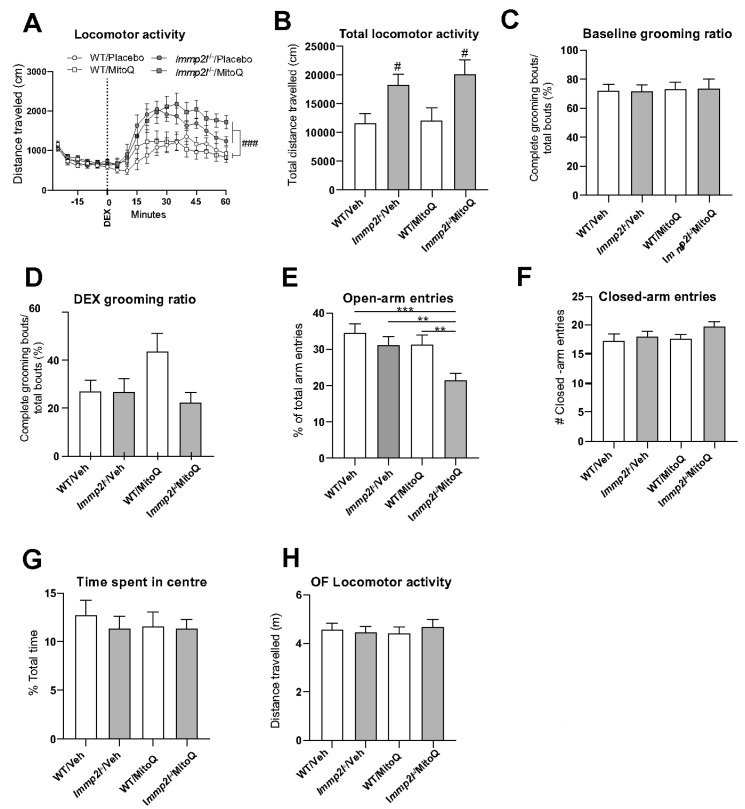
Antioxidant MitoQ does not reverse behavioural changes associated with *Immp2l* KO. (**A**) Mean distance travelled throughout the dexamphetamine locomotor activity test before and after dexamphetamine for *Immp2l*−/− and wild-type mice treated with MitoQ or placebo. (**B**) Mean total distance travelled following dexamphetamine for mice treated with MitoQ or placebo. (**C**) Percentage of completed grooming bouts for mice treated with MitoQ or placebo (n = 12–15/group). (**D**) Percentage of completed grooming bouts for mice treated with MitoQ or placebo in response to dexamphetamine (n = 8/group). (**E**) Ratio of open-arm entries and (**F**) number of closed-arm entries in the elevated-plus maze for mice treated with MitoQ or placebo (n = 15/group). (**G**) Mean time spent in the centre of the open field and (**H**) distance travelled and non-ambulatory movement for mice treated with MitoQ or placebo (n = 11–15/group). *** *p* < 0.001, ** *p* < 0.01 for pairwise comparisons. ### *p* < 0.001, # *p* < 0.05 for genotype differences. Error bars represent SEM.

Oxidative stress has been associated with neurodevelopmental disorders such as ASD, whether as a cause or effect we do not know [1,2,3,4,5,6]. However, given that an earlier truncated *Immp2l*^Tg(Tyr)979Ove^ mouse presented with increased oxidative stress [3] we tested if MitoQ could remediate the dopamine-mediated behaviour in our *Immp2l^KD^* −/− KO mice by assessing dexamphetamine-induced hyperlocomotion. In this study, amphetamine administration increased locomotion in all mice (time main effect: F_(3.23,87.10)_ = 31.61, *p* < 0.001, ε = 0.29; Figure 3A). *Immp2l^KD^* −/− KO mice displayed significantly exacerbated dexamphetamine-induced locomotion compared to WT littermate mice (time × genotype interaction: F_(3.23,87.10)_ = 3.23, *p* = 0.014, ε = 0.29; genotype main effect: F_(1,27)_ = 11.12, *p* = 0.002; Figure 3A,B). MitoQ did not affect amphetamine-induced locomotion in WT mice, but prolonged amphetamine induced locomotion in *Immp2l^KD^* −/− KO mice (time × genotype × treatment interaction: F_(3.23,87.10)_ = 3.85, *p* = 0.010, ε = 0.29). Post-hoc analysis revealed that locomotor activity in *Immp2l*^−/−^ male mice treated with MitoQ (compared to water treated male littermates) was statistically significantly increased (*p* < 0.05) from 30 min post-amphetamine until the end of the experiment (Figure 3A). MitoQ did not affect vertical activity (rearing; *p* > 0.05).

We then sought to extend the characterisation of the behavioural phenotype of *Immp2l^KD^* −/− KO mice for behaviours relevant to ASD or common ASD comorbidities, and examine whether MitoQ may be effective in ameliorating any of these behaviours. Restrictive and repetitive behaviours and movements are a hallmark of ASD. Grooming in mice comprises a complex sequenced structure of movements initiated at the nose and ending in the flank or body. Excessive grooming or disruptions to the normal patterned behaviour has been suggested to represent unwanted repetitive behaviours in mice [75] and can be unmasked by amphetamine administration [86]. To explore whether the increased locomotive movement of *Immp2l^KD^* −/− KO mice was associated with increased grooming bouts or interrupted syntactic grooming sequences, we administered the splash test in mice in the absence and presence of dexamphetamine. Neither genotype nor MitoQ treatment significantly affected the time spent grooming, number of grooming bouts or correct syntactic grooming bouts, regardless of amphetamine treatment (Figure 3C,D and Figure S1). These data demonstrate that Immp2l does not regulate grooming behaviour.

#### 3.4.1. *MitoQ Is Anxiogenic in Immp2l^KD^ −/− KO Mice in the EPM.*

Anxiety and other stress-related disorders commonly co-occur with ASD [87]. To determine if MitoQ modulates approach-avoidance anxiety-like behaviour in *Immp2l^KD^* −/− KO mice, we assessed behaviour in the elevated-plus maze and open field on *Immp2l^KD^* −/− KO vs. wild-type mice treated with MitoQ or placebo. The knockout of Immp2l activity and the treatment with MitoQ each independently reduced the ratio of open-arm entries (genotype main effect: F_(1,56)_ = 8.262, *p* = 0.006; treatment main effect: F_(1,56)_ = 7.850, *p* = 0.007; Figure 3E), which indicates increased avoidance of the aversive area considered to reflect anxiety-relevant behaviour. Post-hoc analysis revealed that *Immp2l^KD^* −/− KO male mice treated with MitoQ showed a statistically significant reduction in the ratio of open-arm entries compared to wild-type male mice treated with MitoQ (*p* = 0.004) and vehicle treated *Immp2l^KD^* −/− KO mice (*p* = 0.004; Figure 3E). This revealed that the impact of MitoQ on this statistical main effect was actually driven by the *Immp2l^KD^* −/− KO group. However, neither the knockout of Immp2l activity or the treatment with MitoQ affected closed-arm entries (Figure 3F), a measure of general locomotor activity confirming that the reduced open-arm entries were not a byproduct of reduced overall activity. Neither *Immp2l^KD^* −/− KO nor MitoQ treatment changed anxiety-like behaviour in the open field as measured by centre-zone time, distance travelled (Figure 3G,H) or rearing (*p* > 0.05 for all). 

#### 3.4.2. *MitoQ Impairs Sensoriomotor Gating Which Is Not Impacted by Immp2l*

**Figure 4:**
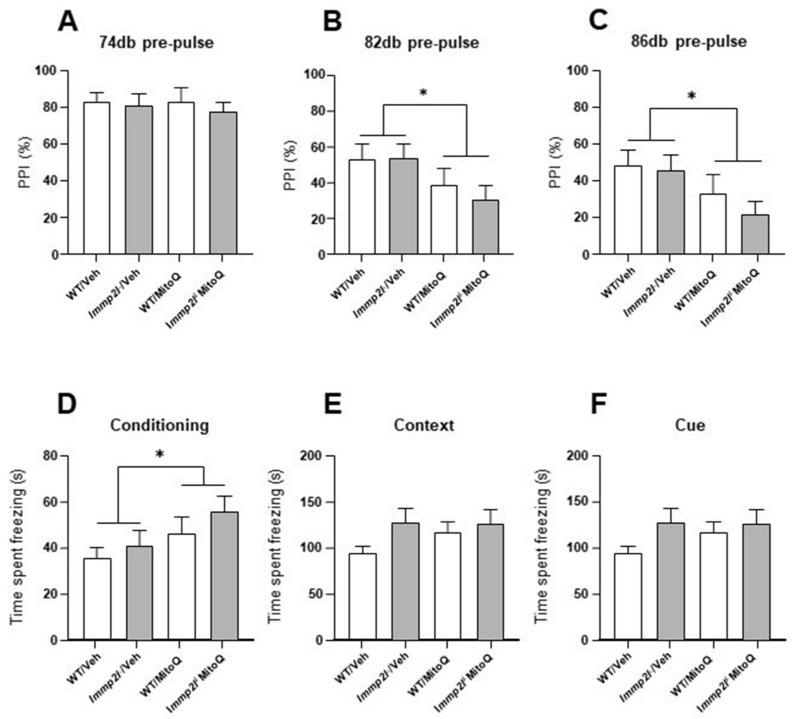
MitoQ impairs sensorimotor gating in both *Immp2l^KD^* −/− KO and wild-type mice but does not affect fear memory. (**A**–**C**) Mean pre-pulse inhibition for *Immp2l*−/− KO and wild-type mice treated with MitoQ or placebo. (**D**–**F**) Freezing behaviour in the fear conditioning test during condition and in response to context and cue induced fear memory. n = 15/group. * *p* < 0.05 for MitoQ treatment differences. Error bars represent SEM.

The open field and elevated plus maze tests measure approach-avoidance conflict in mice to reveal potential differences in anxiety-like behaviours. On the other hand, the acoustic startle response examines domains of sensorimotor gating, deficits of which have been associated with ASD [88]. When preceded by a predictable stimulus, the response to the startle noise is normally reduced, reflecting intact sensorimotor gating. All groups habituated across time to all three pulse intensities (F_(2,112)_ = 162.574, *p* < 0.001). MitoQ impaired pre-pulse inhibition in response to 82 and 86 dB pre-pulse intensities, regardless of genotype (treatment main effect F_(2,112)_ = 10.51, *p* < 0.001) (Figure 4A–C). These data confirm the capacity for the concentrations of MitoQ that reached the brain to modulate brain function and behaviour.

#### 3.4.3. Fear Conditioned Memory Not Affected by Immp2l KO or Treatment with MitoQ

To explore possible cognitive deficits that are associated with ASD [89], we tested fear conditioned learning and memory in *Immp2l^KD^* −/− KO vs. wild-type mice treated with MitoQ or placebo. MitoQ significantly increased freezing during the conditioning phase, regardless of genotype (F_(1,56)_ = 4.69, *p* < 0.05) (Figure 4D), indicating that high doses of MitoQ may alter innate fear-related behaviour. No differences in fear conditioned contextual or cued memory were observed for either genotype or MitoQ treatment (*p* > 0.05 for all; Figure 4E,F). 

### 3.5. Oral Treatment with MitoQ Reaches the Brain

**Figure 5:**
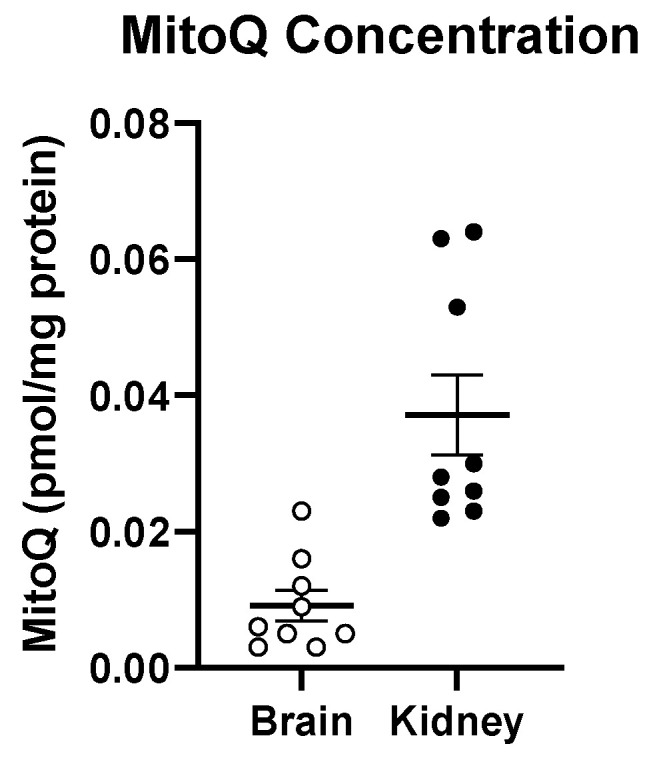
Oral treatment with MitoQ reaches the brain. Concentrations of MitoQ in brain and kidney from mice maintained on MitoQ dissolved in water compared to those on water only from weaning (n = 8–9). Back dots • Refer to individual data points for concentration of MitoQ in kidney and White dots ‘o’ in brain.

We report that MitoQ did not alleviate *Immp2l* KO-mediated behaviour. Furthermore, we confirm that MitoQ did reach the brain of the *Immp2l^KD^* −/− KO mice from where behaviour is governed. We determined the concentration of MitoQ that reached the brain in comparison to a peripheral organ at completion of our studies. We compared MitoQ concentrations in the brain and kidney. The kidney was chosen as a target peripheral organ because it has been shown to be sensitive to oxidative stress and to Immp2l mutations [60,63]. We confirmed that MitoQ was able to cross the blood brain barrier but an approximately 4–6-fold better uptake of MitoQ occurred in the kidney compared with the brain (Figure 5).

### 3.6. Immp2l^KD^ −/− KO Did Not Increase Oxidative Stress

**Figure 6:**
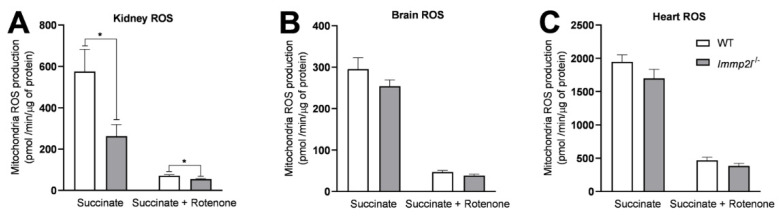
*Immp2l^KD^* −/− KO mice show decreased ROS levels compared to wild-type littermates. (**A**–**C**) Quantification of reactive oxygen species (ROS) generation in mitochondria isolated from kidney, brain and heart (n = 10–18/group) * *p* < 0.05. Error bars represent SEM.

As mentioned earlier, at least 19 different articles have been published describing oxidative stress in the earlier truncated *Immp2l*^Tg(Tyr)979Ove^ mouse developed by Lu et al. [3,28,29,56,57,58,59,60,61,62,63,64,65,66,67,68,69,70,71]. Furthermore, all 19 of these articles noted increased ROS in this single mouse line. These 19 reports in turn formed the basis of our decision to assess whether MitoQ could reverse the behavioural changes that we had observed in the *Immp2l^KD^* −/− KO mouse—which in due course we found not to be the case. Then we explored if the loss of Immp2 activity in *Immp2l^KD^* −/− KO mice was associated with a reduction in ROS—which we found not to be the case. To do this, we quantified ROS generation in mitochondria isolated from the brain, kidney and heart of *Immp2l^KD^* −/− KO mice compared to WT mice. Our newly developed *Immp2l^KD^* −/− KO mouse displayed no evidence of increased ROS in mitochondria isolated from any of the tissues tested. To the contrary, in male *Immp2l^KD^* −/− KO mice, we observed a significant decrease in H_2_O_2_ in the mitochondria from kidney in the presence of succinate (*t*_(26)_ = 2.594, *p* = 0.02) and in the presence of succinate plus rotenone (*t*_(26)_ = 2.449, *p* = 0.02) when compared with wild-type littermates with a similar trend found in the brain of *Immp2l^KD^* −/− KO mice (Figure 6A–C).

### 3.7. Immp2l^KD^ −/− KO Is Not Associated with Neurodegeneration in the Prefrontal Cortex

**Figure 7:**
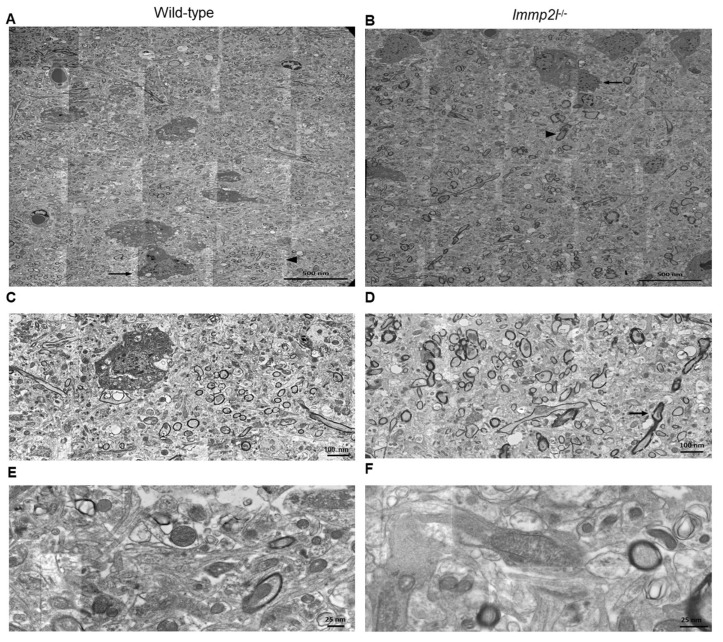
Electron microscopy (EM) micrographs of (**A**,**B**). Sections through the medial prefrontal cortex of adult wild-type and Immp2l^KD^−/− KO male mice, respectively, indicate no evidence of neurodegeneration with myelin sheathed axons (arrowhead) and astrocyte-like cells (long arrow) containing prominent cytoplasmic filament bundles. Myelin sheathing is predominantly round though some irregularity and elongation are seen in Immp2l^KD^−/− KO mouse (**B**). (**C**,**D**) Higher power views of (**A**,**B**), respectively, show normal myelin sheathing surrounding axonal cell processes (arrow). Note: some vacuolation of the myelin sheathing seen in (**C**,**D**) are probably artefacts from the sample preparation. (**E**,**F**) Higher power view of adult male wild-type and Immp2l^KD^−/− KO mice mitochondria in medial prefrontal cortex. EM within cell processes showed regular circular/elliptical outlines (**E**) compared with the regular elliptical outlines (**F**) formed from the double limiting membrane and which contained regularly dispersed internal cristae in both. Note: some image stitching artefacts (fine, straight white lines) are seen in (**E**,**F**). All micrographs were scanned at 5 nm resolution.

We assessed our new *Immp2l^KD^* −/− KO mice for any oxidative stress-related phenotypes that may influence behaviour; namely age-related ataxia and neurodegeneration. In this investigation we found that *Immp2l^KD^* −/− KO female and male mice showed no evidence of ataxia beyond 18 months of age (see Results Section 3.1). Upon dissection of the brain of male and female *Immp2l^KD^* −/− KO mice (n = 30), all regions of the brain examined appeared in excellent condition with no evidence of overt neurodegeneration [55]. We used electron microscopy (EM) to interrogate high-resolution images of the medial prefrontal cortex of male adult mice for evidence of neurodegeneration. High-resolution EM images provided no evidence of neurodegeneration in *Immp2l^KD^* −/− KO male mice (Figure 7A–F). More extensive immunohistochemical analyses further confirmed that there was no evidence of neurodegeneration in other dopamine related regions of the brain of *Immp2l^KD^* −/− KO male mice nor was there any reduction in neuronal number in these regions of the brain tested [55]. 

### 3.8. Structural Analysis of Heterodimerisation between Immp1l and ‘Truncated Immp2l’ 

The present study reports dramatic phenotypic differences between the *Immp2l^KD^−/−* KO mouse and the earlier truncated Immp2l^Tg(Tyr)979Ove^ mouse model reported by Lu et al. in 2008 [3]. This, despite the inactivity of the Immp2l peptidase in both mouse models [3]. These findings indicate that some difference other than the loss of Immp2l peptidase activity is the basis of these phenotypic differences. In this respect, the most prominent difference between the two models is the expression of a truncated form of Immp2l in the earlier *Immp2l^Tg(Tyr)979Ove^* mouse model in association with extensive oxidative stress phenotypes [3]. This truncation removed a large part of the C-terminus leaving intact the N-terminus of Immp2l that directs the transit of Immp2l to the inner mitochondrial membrane (IMM) [3], thus providing a pathway for ‘truncated Immp2l’ to reach the IMM where electron transport and ROS levels are largely regulated. 

**Figure 8:**
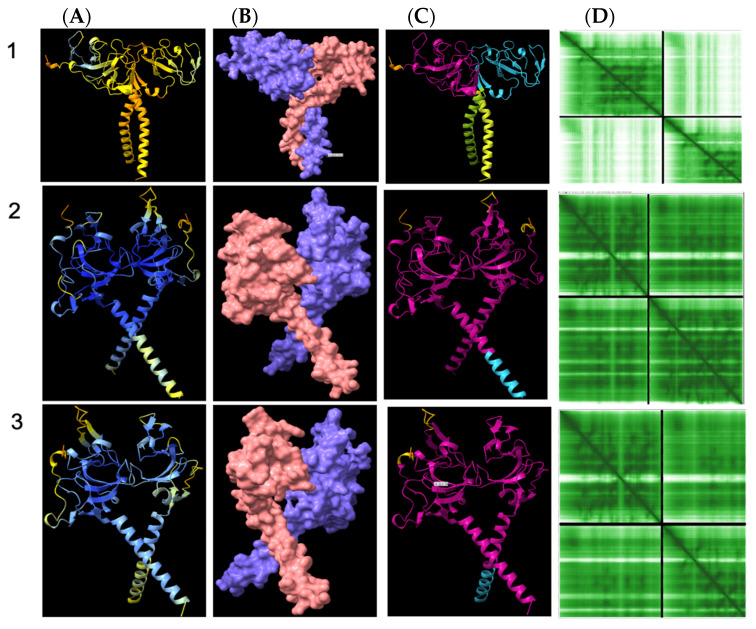
Cartoon representation of AlphaFold2-Multimer high-accuracy prediction of Immp1l-Immp2l heterodimeric structures. AlphaFold2-Multimer [82] was used to predict dimerisation with predicted alignment error (PAE) plots visualised through ChimeraX. Rows: **1**. Heterodimerisation between Immp2l and ‘Truncated Immp2l’ (pink); **2.** Heterodimerisation between Immp1l and Immp2l (pink); **3.** Heterodimerisation between Immp1l and ‘Truncated Immp2l’ (pink). Columns: (**A**) Monomer reliability colour coded where dark blue > 93% confidence; lighter blue 90–93%; cyan 83–89%; green 71–82%; yellow 60–70%; orange 56–59% and red < 56% confidence; (**B**) surface structure; (**C**) dimer PAE colour comparison; (**D**) PAE plots. For PAE plots, higher green colour intensity depicts higher confidence. Upper left and lower right quadrants represent monomer confidence whereas lower left and upper right quadrants depict dimerization confidence.

Immp1l is a close homologue of Immp2l and the amino acid sequences of both are conserved between E. coli, yeast, mouse and human [90]. Moreover, in the IMM of yeast, Imp2 (homologue of Immp2l) heterodimerises with Imp1 (homologue of Immp1l) to form the IMP complex [85,91]. The loss of Imp2 destabilises this IMP complex, presumably through loss of Imp2-Imp1 heterodimerisation complexes. This destabilisation can in turn be reversed by the expression of mouse Immp2l in yeast, presumably through Immp2l-Imp1 heterodimerisation [92] thereby demonstrating functional complementation between yeast Imp2 and mouse Immp2l. Using the AlphaFold2-Multimer program visualised through the ChimeraX quantitative assessment program, we compared the quality and confidence of Immp2l-Immp1l heterodimerisation using Predicted Alignment Error (PAE) analysis [84]. Despite not knowing the precise C-terminal extension sequence of the ‘Truncated-Immp2l’ reported by Lu et al. [3], AlphaFold2-Multimer analysis predicted with high confidence that ‘Truncated-Immp2l’ heterodimerises with Immp1l (Figure 8—Row 3). In comparable fashion AlphaFold2-Multimer predicted with high confidence the heterodimerisation between ‘Full-length Immp2l’ and Immp1l (Figure 8—Row 2) which had been experimentally demonstrated for the respective homologues (Imp2 and Imp1) in yeast [85,91]. This was in stark contrast with the very low predicted confidence of heterodimerisation between ‘Truncated-Immp2l’ and ‘Full-length Immp2l’ (Figure 8—Row 1). This high predicted confidence for the heterodimerisation of ‘Truncated-Immp2l’ with Immp1l (Figure 8—Row 3) in turn expands the potential for dominant negative gain-of-function affects arising from the C-terminal truncated form of Immp2l expressed in the earlier Immp2l^Tg(Tyr)979Ove^ mouse model [3]. 

## 4. Discussion

**Figure 9:**
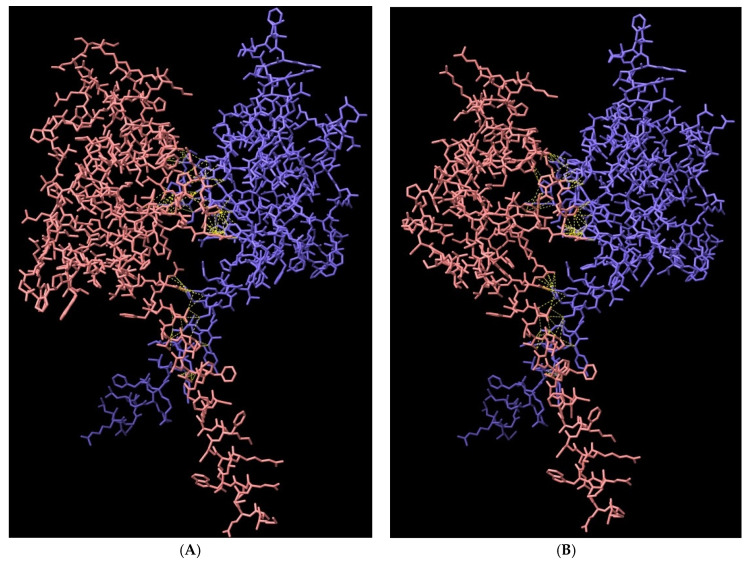
Cartoon representation of AlphaFold2-Multimer high-accuracy prediction of heterodimeric connections between (**A**) Immp1l and Immp2l (pink); (**B**) Immp1l and ‘Truncated-Immp2l’ (pink) [3] with 110 and 94 predicted connections (yellow), respectively, most of which occur in comparable positions between the respective heterodimers.

**Figure 10:**
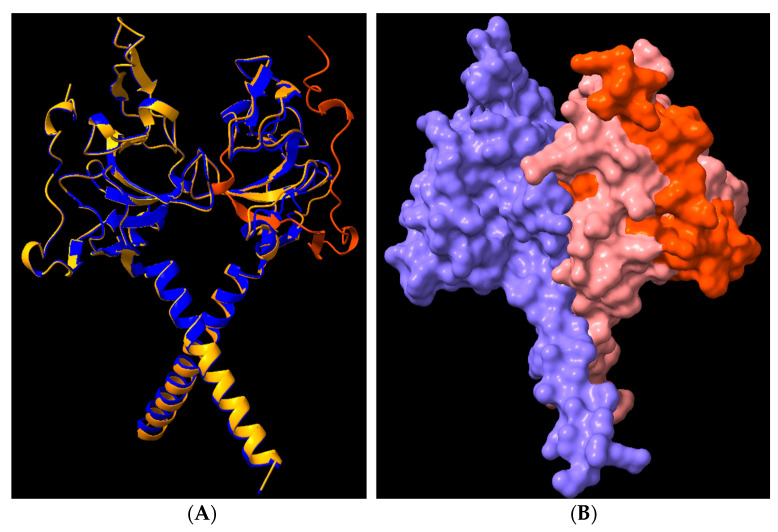
Cartoon representation of AlphaFold2-Multimer high-accuracy prediction of heterodimerisation between (**A**,**B**). Immp1l (**left**) and Immp2l (**right**) with the C-terminal truncated region of Immp2l that was deleted in the earlier *Immp2l^Tg(Tyr)979Ove^* mouse model coloured orange [3].

The 7q31 chromosome locus has been repeatedly linked to ASD inheritance and fine mapping identified *IMMP2L* as the gene linked to ASD at 7q31 (Table 1) [9,10,11,12,13,23]. However, the precise biological basis of this linkage has yet to be determined and it remains unknown whether it is representative of loss or gain of IMMP2L function and how that may positively or negatively affect behaviour. High relative incidence of heterozygous intragenic deletions within *IMMP2L* have also been identified in ASD and in normal ‘neurotypical’ populations (Table 2 and Table 3) [40,40,44,47] casting uncertainty on their role if any in ASD or other related neurodevelopmental disorders such as GTS and ADHD (Table 1, Table 2 and Table 3). Albeit the AlphaFold2-Multimer high-accuracy predictive analysis performed in this study (Figure 8) does raise the spectre of possible gain-of-function dominant negative oxidative stress effects arising from that subset of ASD-related *IMMP2L* intragenic deletions that give rise to C-terminal-truncated-IMMP2L variants (see Table 2 and Table 3 and Figure 8, Figure 9 and Figure 10) [3,84]. In this respect we found no evidence for any of the core symptoms of ASD associated with the loss of Immp2l activity in *Immp2l*^KD^ −/− or −/+ KO mice [54]. However, this does not discount the possibility that ASD-like behaviours may develop due to gain-of-function effects arising from truncated forms of Immp2l like that caused by the intragenic deletion in *Immp2l* in the earlier *Immp2l*^Tg(Tyr)979Ove^ mouse model (Figure 8, Figure 9 and Figure 10). Unfortunately, in this respect *Immp2l*^Tg(Tyr)979Ove^ mice cannot be tested for behavioural changes or for any possible behavioural benefits from MitoQ or SKQ1 treatment due their having been bred from a blind mouse strain [3]. 

In contrast, *Immp2l^KD^* −/− KO mice present with increased auditory stimulus-driven instrumental behaviour [55] and increased amphetamine-induced locomotion (Figure 2) [54] and an increase in freezing behaviour in the conditioning phase of the fear conditioning test (Figure 4D) which is suggestive of possible improved learning (see Table 2). Notwithstanding the biological source of these behavioural changes is unknown and if they in any way relate to the core features of ASD, which otherwise form the basis of the ASD linkage studies cited above, is uncertain [54]. 

Due to reports of oxidative stress in an earlier truncated *Immp2l*^Tg(Tyr)979Ove^ mouse model [3,28,29,56,57,58,59,60,61,62,63,64,65,66,67,68,69,70,71], we investigated whether oxidative stress was evident in our new *Immp2l^KD^* −/− KO mouse and/or whether it had a role in their enhanced amphetamine-induced locomotion. Our Western blot analyses demonstrate that the *Immp2l^KD^* −/− KO mouse [53] is devoid of Immp2l peptidase activity with no evidence of processing of two of the known Immp2l substrates Cyc1 and Gpd2 (Figure 1). Furthermore, we demonstrate that the loss of this Immp2l peptidase activity was associated with increased sensitivity to dexamphetamine, presumably through enhanced dopamine responses (Figure 2). Using this viable *Immp2l^KD^* −/− KO mouse model [54], we then found that treatment with the antioxidant MitoQ, which is proven to effectively target mitochondria-induced oxidative stress, did not reverse or moderate the behavioural changes displayed by these mice (Figure 3). Consistent with this finding, we found that ROS levels were not increased but decreased in the *Immp2l^KD^* −/− KO mouse (Figure 6) which was consistent with the absence of any ROS-mediated oxidative stress-related phenotypes in these mice (Figure 7 and Table 4 and Table 5) [55]. These findings demonstrate that loss of Immp2l activity does not increase oxidative stress and that antioxidant treatment using MitoQ does not appear to have any therapeutic value in this context.

In *Immp2l^KD^* −/− KO mice, both Cyc1 and Gpd2 retain their mitochondrial signal peptides within the mitochondria. This confirmed the loss of Immp2l activity in *Immp2l^KD^* −/− KO mice and may also help explain the decrease in ROS in these mice and their dopamine-mediated behavioural dysregulation, but how these two may be related is unknown. The findings in this and other studies further indicate that the Immp2l-mediated dopamine dysregulation is unlikely to be due to neurodegeneration [55,56,93,94]. Moreover, the EM performed on the prefrontal cortex in this study (Figure 7) together with the extensive immunohistochemical investigation of other dopamine associated brain regions by Leung et al. [55] does appear to confirm this hypothesis [55]. Furthermore, our investigations indicate that dexamphetamine/dopamine-mediated behaviours are most affected by the KO of *Immp2l* because behaviours that are far less governed by dopamine were largely unaffected, such as conditioned fear and elevated plus maze behaviour. However, PPI, which is also mediated by dopaminergic neurotransmission [95], was not impacted by the loss of Immp2l activity. This further suggests that Immp2l effects on the dopamine system are limited and selective and may only affect specific dopaminergic subsystems [96]. Indeed, the dopamine mesolimbic circuits that mediate PPI are likely to differ to those that mediate the locomotor activity described above [97], and similarly different parts of the striatum respond to amphetamine to regulate locomotion versus stereotypy [98]. Notwithstanding, both ASD and GTS have been linked to altered dopaminergic neurotransmission and antipsychotic drugs are used in their treatment, albeit, not for the core symptoms of ASD which are also shared to varying degrees with GTS [47,99]. Together, these findings demonstrate the validity of the *Immp2l^KD^* −/− KO mouse model in investigating the contribution of IMMP2L to clinically relevant features associated with oxidative stress-free dopamine modulated brain function and behaviour. 

The findings of this study suggest that oxidative stress does not mediate the increased dexamphetamine-induced locomotion observed in the *Immp2l^KD^* −/− KO mouse, and that the oral antioxidant MitoQ shows no therapeutic benefit in this context. This contrasts with the earlier truncated *Immp2l*^Tg(Tyr)979Ove^ mouse that suggested loss of Immp2l activity was associated with an increase in ROS production and oxidative stress phenotypes including oxidative stress in the brain causing ataxia and neurodegeneration [3,28,29,56,57,58,59,60,61,62,63,64,65,66,67,68,69,70,71]. Moreover, ROS has been implicated as a mediator of the effects of amphetamine on locomotion [100]. However, our data provide convincing evidence that ROS-mediated oxidative stress is not evident in the *Immp2l^KD^* −/− KO mouse model and that it is not involved in the behavioural changes that result from the loss of Immp2l activity in this model. Firstly, we found no evidence of oxidative stress-associated phenotypes in the *Immp2l^KD^* −/− KO mouse line including no infertility in females or males (Table 4 and Table 5), nor was there age-related ataxia or any overt or microscopic evidence of neurodegeneration [55]. Secondly, chronic MitoQ, a proven mitochondrial targeted antioxidant treatment, was unable to reverse or moderate the behavioural consequences of *Immp2l^KD^* −/− KO. Thirdly, we found no evidence of increased H_2_O_2_/superoxide levels in *Immp2l^KD^* −/− KO tissues, including the brain. To the contrary, ROS levels were decreased in the kidney of *Immp2l^KD^* −/− KO mice with a similar trend in the brain indicative of an antioxidant-like phenotype. Fourthly, there was evidence of additive antioxidant effects between the *Immp2l^KD^* −/− KO genotype and the MitoQ on anxiety-relevant behaviour in the elevated plus maze (Figure 3E) and in time spent freezing during the training phase of the fear conditioning test (Figure 4D). 

MitoQ was chosen for its specificity and proven capacity to target mitochondrial-induced oxidative stress in human and mouse [30]. We considered the possibility that the dose of MitoQ used in this study was not sufficient to reach the brain in concentrations that could plausibly impact behaviour but believe this is unlikely for the following reasons: (1) as far as we are aware, this study utilised the highest dose and longest treatment of oral MitoQ in animals compared with any other study, while other studies using lower doses have reported effects [33,34]; (2) we confirmed the capacity of our high MitoQ regimen to reach the brain (Figure 5); and (3) that MitoQ treatment affected several behaviours, confirming that MitoQ was able to influence brain-directed function and behaviour but not the increase in amphetamine/dopamine-mediated behaviour (Figure 3E and Figure 4B–D). 

The molecular basis for the differences in oxidative stress effects between our new *Immp2l^KD^* −/− KO mouse model and the earlier truncated *Immp2l*^Tg(Tyr)979Ove^ mouse is uncertain [3,28,29,56,57,58,59,60,61,62,63,64,65,66,67,68,69,70,71]. It is possible that the different strains of mice used had sufficiently different genetic backgrounds to differentially regulate gene-gene interactions and oxidative stress (i.e., C57BL/6J versus FVB/N mouse strains). Alternatively, the complex genotype of the *Immp2l*^Tg(Tyr)979Ove^ mouse inclusive of the integration of a foreign transgene and an unexpected genomic deletion within the final intron of the *Immp2l* gene may have been implicated. One possibility is that of transgene-mediated tyrosinase expression. Another more likely scenario is that the earlier truncated *Immp2l^Tg(Tyr)979Ove^* mouse model [3,28,29,56,57,58,59,60,61,62,63,64,65,66,67,68,69,70,71] was not a clean KO but rather it expressed a largely intact yet truncated form of Immp2l that may have had unrecognised gain-of-function dominant negative effects on mitochondrial function with oxidative stress effects (Figure 8 and Figure 9). By comparison, oxidative stress effects appeared to be totally absent from our more recent *Immp2l^KD^* −/− KO mouse which presented with what could be considered a reversed antioxidant-like phenotype with a decrease in ROS and some behaviours that mimic those associated with the antioxidant MitoQ, including reduced number of open-arm entries (genotype main effect: F_(1,56)_ = 8.262, *p* = 0.006; treatment main effect: F_(1,56)_ = 7.850, *p* = 0.007; Figure 3E). As such, not only were oxidative stress phenotypes absent from the *Immp2l^KD^* −/− KO mouse reported in this study, but that a reverse antioxidant-like molecular and behavioural phenotype was evident which strongly suggests that the oxidative stress phenotypes of the earlier truncated *Immp2l^Tg(Tyr)979Ove^* mouse may have been the result of dominant negative effects arising from its expression of the truncated form of Immp2l (Figure 8 and Figure 9) and not its associated loss of Immp2l activity. However, more research is needed to determine precisely why these two Immp2l mouse models differ in mitochondrial function, but our findings demonstrate that Immp2l knockout-related behavioural changes are not dependent on increases in oxidative stress (Figure 1 and Table 1, Table 2 and Table 3) [3,9,11,12,13,20,26,28,29,56,57,58,59,60,61,62,63,64,65,66,67,68,69,70,71,101,102].

A number of the results from the present study are limited in their scope and require further investigation. Firstly, our electron microscopy found no evidence for neurodegeneration in the medial prefrontal cortex. Follow-up immunohistochemical studies supported this finding [55], albeit broader use of electron microscopy would allow for more in depth investigation of other dopamine rich brain regions to discount region-specific oxidative stress. Secondly, the surprising decrease in ROS in the *Immp2l^KD^* −/− KO mouse was consistent with the absence of any overt oxidative stress phenotypes including no infertility nor ataxia and this was confirmed by the inability of the antioxidant MitoQ to reverse the associated behavioural changes. As such, the mechanism causing the decrease in ROS in the *Immp2l^KD^* −/− KO mouse is worthy of further investigation as are its effects on behaviour and aging. Thirdly, MitoQ affected time spent freezing during the training phase of the fear conditioning test in WT controls as well as in *Immp2l^KD^* −/− KO mice. Given that this later finding occurred only during the training phase may indicate that a reduction in oxidative stress, even from baseline levels, aids in fear-conditioned learning (Figure 4D) and as such the possibility of enhanced learning in *Immp2l^KD^* −/− KO mice should be explored further [55]. Finally, the behavioural deficits of the *Immp2l^KD^* −/− *KO* mouse were sensitive to dexamphetamine which suggests that the loss of Immp2l leads to brain hyperdopaminergia and this needs to be tested further. The role of truncated Immp2l variants in behaviour could be evaluated further on a C57BL/6J background. 

## 5. Conclusions

In summary, we demonstrate the validity of our new *Immp2l^KD^* −/− KO mouse model for investigating the impact of Immp2l KO in the pathophysiology of brain and behaviour. Moreover, we demonstrate that the *Immp2l^KD^* −/− KO mouse model is associated with lower ROS levels and an antioxidant-like phenotype associated with behavioural changes including increased auditory stimulus-driven instrumental behaviour [55], increased amphetamine-induced locomotion (Figure 2) [54] and an increase in freezing behaviour in the conditioning phase of the fear conditioning test (Figure 4D) suggestive of possible improved learning in the *Immp2l^KD^* −/− KO mouse. Finally, we conclude that oxidative stress is not driving the dopamine-related behaviour associated with the loss of Immp2l activity in the *Immp2l^KD^* −/− KO mouse model and that antioxidant treatment using MitoQ does not appear to have any therapeutic value in this context. The exploration of the mechanistic role of Immp2l activity in the dopamine mediated regulation of behaviour is anticipated in future studies.

## Figures and Tables

**Table 1:** Multiple ASD genome-wide association studies link the *IMMP2L* gene locus on 7q31.

Association	Locus	Reference
GWAS	7q31	IMGSAC 1998 [9]
GWAS	7q31	Ashley-Kock et al., 1999 [17]
GWAS	7q31	Barrett et al., 1999 [10]
GWAS	7q31	MGSAC 2001 [18]
GWAS	7q31	Shao et al., 2002 [11]
GWAS	7q31	Auranen et al., 2002 [19]
GWAS	7q31	Lamb et al., 2005 [20]
GWAS	7q31	Schellenberg et al., 2006 [12]
Meta-analysis	7q31 (4 ASD GWAS)	Buxbaum et al., 2001 [21]
Meta-analysis	7q31 (9 ASD GWAS)	Badner et al., 2002 [22]
Multiplex	7q31	Trikalinos et al., 2006 [26]
High Density Analysis	IMMP2L SNP rs12537269	Maestrini et al., (IMGSAC) [13]
Haplotype Sharing	IMMP2L	Casey et al., [23]
e-TDT SNP Analysis	IMMP2L in GTS SNP D7S1516	Diaz-Anzaldua et al., 2004 [24]
e-TDT SNP Analysis	IMMP2L in GTS SNP rs7795011	Pagliaroli et al., 2020 [27]
Methylation analysis	IMMP2L	Zhang et al., 2022 [25]

**Table 2:** Human ASD case reports with *IMMP2L* deletions.

Study	Deletion	Gender	Phenotypes
Maestrini [13]	Exonic	Male 13-0577-003	ASD
	Exonic	Male 15-0086-003	ASD
Maestrini and Pagnamenta [13,39]	Exonic	Male 15-0084	ASD
Maestrini [13]	*IMMP2L* Linkage	Paternal	ASD
Bertelsen et al., 2014 [40]	Exonic	Male	ASD, GTS, ADHD, Asperger
Zhang et al., 2017 [41]	Exonic	Male	ASD, Speech Delay, Echo, MR
	Exonic	Male	ASD, Speech Delay, Echo, MR
	Exonic	Male	ASD, Speech Delay, Echo MR
Gimelli et al., 2014 [42]	Exonic	Male	Autistic, Speech Delay, ADHD
Baldan 2018 [38]	Intronic	Male	ASD, Speech Delay
	Exonic	Male	ASD, Speech Delay
Leblond et al., 2019 [37]	Exonic	-	ASD
Qaiser et al., 2021 [46]	Exonic	-	ASD
Vinas-Jornet 2018 [45]	Exonic	-	ASD
*ASD Overlap Disorders*			
Jang et al., 2019 [43]	3 × Exonic	-	Speech Delay, MR, Dev Delay
Gimelli et al., 2014 [42]	Exonic	Male	Speech Delay
	Exonic	Male	Speech Delay
	Exonic	Female	Unaffected
Bertelsen et al., 2014 [40]	6 × Exonic	Males x 6	GTS, ADHD/OCD
Elia et al., 2010 [44]	Exonic	Male	ADHD

**Table 3:** *IMMP2L* deletions and breakpoints in Tourette syndrome (GTS).

Study	Type Association	Phenotypes	Gender	Family Phenotypes
Cytogenetic	Near Breakpoint	GTS, Temper	Male	Tics and OCD [48]
Cytogenetic	Disrupted	GTS	Male	[49]
CNV/LOH	*IMMP2L* Deleted	GTS, Speech delay	Male	[50]
	+ *FOXP2* Deleted	+ Verbal Dyspraxia		
CNV/LOH	Exonic Deletion	GTS, ADHD	Male	Dyslexia, Temper [40]
CNV/LOH	Exonic Deletion	GTS, ADHD	Male	Tics and OCB [40]
CNV/LOH	Exonic Deletion	GTS, ADHD, Asperger	Male	Unaffected [40]
CNV/LOH	Exonic Deletion	GTS, OCD	Male	OCB, Stuttering [40]
CNV/LOH	Exonic Deletion	GTS	Male	Unaffected [40]
CNV/LOH	Exonic Deletion	GTS, ADHD, OCD	Male	Stubbornness [40]
CNV/LOH	Exonic Deletion	GTS, ADHD	Male	Unaffected [40]
eTDT SNP	Biased Transmission	GTS, ADHD, and OCD	-	Biased Transmission [24]

**Table 4:** Litter size and sex distribution of offspring from *Immp2l^KD^* −/− KO (Homo), heterozygous (Het), and wild-type (WT) breeding pairs.

Pups	Homo × Homo	Female Homo × Male WT	Het × Het
Live Born (av/litter)	4.7	7.3	6.7
Weaned	4.0	6.8	6.2
Males	56%	50%	56%
Females	44%	50%	44%

**Table 5:** Sex and genotype distribution of offspring from *Immp2l^KD^* −/+ KO heterozygous X heterozygous mating.

Sex	Genotype	Percentage
Female	Homozygous	9%
Female	Heterozygous	24%
Female	Wild-type	11%
Male	Homozygous	15%
Male	Heterozygous	28%
Male	Wild-type	13%

## Data Availability

Not applicable.

## Supplementary Materials

The following supporting information can be downloaded at https://www.mdpi.com/article/10.3390/genes14091717/s1. Figure S1: Grooming behaviour in Immp2lKD −/− KO mice treated with MitoQ. A–D. Mean time spent grooming, number of grooming bouts, number of completed grooming bouts with the appropriate nose-to-flank sequence, and percentage of completed grooming bouts for Immp2lKD −/− KO and wild-type mice treated with MitoQ or placebo (n = 12-15/group). E–H. Mean time spent grooming, number of grooming bouts, number of completed grooming bouts with the appropriate nose-to-flank sequence, and percentage of completed grooming bouts for Immp2lKD −/− KO and wild-type mice treated with MitoQ or placebo in response to dexamphetamine (n = 8/group). Error bars represent SEM.

## References

[1] Carpita B., Muti D., Dell’Osso L. (2018). Oxidative Stress, Maternal Diabetes, and Autism Spectrum Disorders. Oxid. Med. Cell. Longev..

[2] Manivasagam T., Arunadevi S., Essa M.M., SaravanaBabu C., Borah A., Thenmozhi A.J., Qoronfleh M.W. (2020). Role of Oxidative Stress and Antioxidants in Autism. Adv. Neurobiol..

[3] Lu B., Poirier C., Gaspar T., Gratzke C., Harrison W., Busija D., Matzuk M.M., Andersson K.E., Overbeek P.A., Bishop C.E. (2008). A mutation in the inner mitochondrial membrane peptidase 2-like gene (Immp2l) affects mitochondrial function and impairs fertility in mice. Biol. Reprod..

[4] Morimoto M., Hashimoto T., Kitaoka T., Kyotani S. (2018). Impact of Oxidative Stress and Newer Antiepileptic Drugs on the Albumin and Cortisol Value in Severe Motor and Intellectual Disabilities with Epilepsy. J. Clin. Med. Res..

[5] Joseph N., Zhang-James Y., Perl A., Faraone S.V. (2015). Oxidative Stress and ADHD: A Meta-Analysis. J. Atten. Disord..

[6] Mousavinejad E., Ghaffari M.A., Riahi F., Hajmohammadi M., Tiznobeyk Z., Mousavinejad M. (2018). Coenzyme Q(10) supplementation reduces oxidative stress and decreases antioxidant enzyme activity in children with autism spectrum disorders. Psychiatry Res..

[7] Kern J.K., Geier D.A., King P.G., Sykes L.K., Mehta J.A., Geier M.R. (2015). Shared Brain Connectivity Issues, Symptoms, and Comorbidities in Autism Spectrum Disorder, Attention Deficit/Hyperactivity Disorder, and Tourette Syndrome. Brain Connect..

[8] Clarke R.A., Lee S., Eapen V. (2012). Pathogenetic model for Tourette syndrome delineates overlap with related neurodevelopmental disorders including Autism. Transl. Psychiatry.

[9] (1998). A full genome screen for autism with evidence for linkage to a region on chromosome 7q. International Molecular Genetic Study of Autism Consortium. Hum. Mol. Genet..

[10] Barrett S., Beck J.C., Bernier R., Bisson E., Braun T.A., Casavant T.L., Childress D., Folstein S.E., Garcia M., Gardiner M.B. (1999). An autosomal genomic screen for autism. Collaborative linkage study of autism. Am. J. Med. Genet..

[11] Shao Y., Wolpert C., Raiford K., Menold M., Donnelly S., Ravan S., Bass M., McClain C., von Wendt L., Vance J. (2002). Genomic screen and follow-up analysis for autistic disorder. Am. J. Med. Genet..

[12] Schellenberg G.D., Dawson G., Sung Y.J., Estes A., Munson J., Rosenthal E., Rothstein J., Flodman P., Smith M., Coon H. (2006). Evidence for multiple loci from a genome scan of autism kindreds. Mol. Psychiatry.

[13] Maestrini E., Pagnamenta A.T., Lamb J.A., Bacchelli E., Sykes N.H., Sousa I., Toma C., Barnby G., Butler H., Winchester L. (2010). High-density SNP association study and copy number variation analysis of the AUTS1 and AUTS5 loci implicate the IMMP2L-DOCK4 gene region in autism susceptibility. Mol. Psychiatry.

[14] Chakrabarti S., Fombonne E. (2005). Pervasive developmental disorders in preschool children: Confirmation of high prevalence. Am. J. Psychiatry.

[15] Fombonne E. (2005). Epidemiology of autistic disorder and other pervasive developmental disorders. J. Clin. Psychiatry.

[16] Baird G., Simonoff E., Pickles A., Chandler S., Loucas T., Meldrum D., Charman T. (2006). Prevalence of disorders of the autism spectrum in a population cohort of children in South Thames: The Special Needs and Autism Project (SNAP). Lancet.

[17] Ashley-Koch A., Wolpert C.M., Menold M.M., Zaeem L., Basu S., Donnelly S.L., Ravan S.A., Powell C.M., Qumsiyeh M.B., Aylsworth A.S. (1999). Genetic studies of autistic disorder and chromosome 7. Genomics.

[18] International Molecular Genetic Study of Autism Consortium (IMGSAC) (2001). A genomewide screen for autism: Strong evidence for linkage to chromosomes 2q, 7q, and 16p. Am. J. Hum. Genet..

[19] Auranen M., Vanhala R., Varilo T., Ayers K., Kempas E., Ylisaukko-Oja T., Sinsheimer J.S., Peltonen L., Järvelä I. (2002). A genomewide screen for autism-spectrum disorders: Evidence for a major susceptibility locus on chromosome 3q25-27. Am. J. Hum. Genet..

[20] Lamb J.A., Barnby G., Bonora E., Sykes N., Bacchelli E., Blasi F., Maestrini E., Broxholme J., Tzenova J., Weeks D. (2005). Analysis of IMGSAC autism susceptibility loci: Evidence for sex limited and parent of origin specific effects. J. Med. Genet..

[21] Buxbaum J.D., Silverman J.M., Smith C.J., Kilifarski M., Reichert J., Hollander E., Lawlor B.A., Fitzgerald M., Greenberg D.A., Davis K.L. (2001). Evidence for a susceptibility gene for autism on chromosome 2 and for genetic heterogeneity. Am. J. Hum. Genet..

[22] Badner J.A., Gershon E.S. (2002). Regional meta-analysis of published data supports linkage of autism with markers on chromosome 7. Mol. Psychiatry.

[23] Casey J.P., Magalhaes T., Conroy J.M., Regan R., Shah N., Anney R., Shields D.C., Abrahams B.S., Almeida J., Bacchelli E. (2012). A novel approach of homozygous haplotype sharing identifies candidate genes in autism spectrum disorder. Hum. Genet..

[24] Díaz-Anzaldúa A., Joober R., Rivière J.-B., Dion Y., Lespérance P., Chouinard S., Richer F., Rouleau G.A., Montreal Tourette Syndrome Study Group (2004). Association between 7q31 markers and Tourette syndrome. Am. J. Med. Genet. A.

[25] Zhang B., Hu X., Li Y., Ni Y., Xue L. (2022). Identification of methylation markers for diagnosis of autism spectrum disorder. Metab. Brain Dis..

[26] Trikalinos T.A., Karvouni A., Zintzaras E., Ylisaukko-oja T., Peltonen L., Järvelä I., Ioannidis J.P.A. (2006). A heterogeneity-based genome search meta-analysis for autism-spectrum disorders. Mol. Psychiatry.

[27] Pagliaroli L., Vereczkei A., Padmanabhuni S.S., Tarnok Z., Farkas L., Nagy P., Rizzo R., Wolanczyk T., Szymanska U., Kapisyzi M. (2020). Association of Genetic Variation in the 3’UTR of LHX6, IMMP2L, and AADAC With Tourette Syndrome. Front. Neurol..

[28] Jiang Y., Liu C., Lei B., Xu X., Lu B. (2018). Mitochondria-targeted antioxidant SkQ1 improves spermatogenesis in Immp2l mutant mice. Andrologia.

[29] Liu C., Li X., Lu B. (2016). The Immp2l mutation causes age-dependent degeneration of cerebellar granule neurons prevented by antioxidant treatment. Aging Cell.

[30] Tauskela J.S. (2007). MitoQ—A mitochondria-targeted antioxidant. IDrugs.

[31] Antonenko Y.N., Avetisyan A.V., Bakeeva L.E., Chernyak B.V., Chertkov V.A., Domnina L.V., Ivanova O.Y., Izyumov D.S., Khailova L.S., Klishin S.S. (2008). Mitochondria-targeted plastoquinone derivatives as tools to interrupt execution of the aging program. 1. Cationic plastoquinone derivatives: Synthesis and in vitro studies. Biochemistry.

[32] Rivera-Barahona A., Alonso-Barroso E., Pérez B., Murphy M.P., Richard E., Desviat L.R. (2017). Treatment with antioxidants ameliorates oxidative damage in a mouse model of propionic acidemia. Mol. Genet. Metab..

[33] Li G., Chan Y.L., Sukjamnong S., Anwer A.G., Vindin H., Padula M., Zakarya R., George J., Oliver B.G., Saad S. (2019). A Mitochondrial Specific Antioxidant Reverses Metabolic Dysfunction and Fatty Liver Induced by Maternal Cigarette Smoke in Mice. Nutrients.

[34] Sukjamnong S., Chan Y.L., Zakarya R., Nguyen L.T., Anwer A.G., Zaky A.A., Santiyanont R., Oliver B.G., Goldys E., Pollock C.A. (2018). MitoQ supplementation prevent long-term impact of maternal smoking on renal development, oxidative stress and mitochondrial density in male mice offspring. Sci. Rep..

[35] Robertson M.M., Eapen V., Singer H.S., Martino D., Scharf J.M., Paschou P., Roessner V., Woods D.W., Hariz M., Mathews C.A. (2017). Gilles de la Tourette syndrome. Nat. Rev. Dis. Primers.

[36] Gabriels R.L., Cuccaro M.L., Hill D.E., Ivers B.J., Goldson E. (2005). Repetitive behaviors in autism: Relationships with associated clinical features. Res. Dev. Disabil..

[37] Leblond C.S., Cliquet F., Carton C., Huguet G., Mathieu A., Kergrohen T., Buratti J., Lemière N., Cuisset L., Bienvenu T. (2019). Both rare and common genetic variants contribute to autism in the Faroe Islands. NPJ Genom. Med..

[38] Baldan F., Gnan C., Franzoni A., Ferino L., Allegri L., Passon N., Damante G. (2018). Genomic Deletion Involving the IMMP2L Gene in Two Cases of Autism Spectrum Disorder. Cytogenet. Genome Res..

[39] Pagnamenta A.T., Bacchelli E., de Jonge M.V., Mirza G., Scerri T.S., Minopoli F., Chiocchetti A., Ludwig K.U., Hoffmann P., Paracchini S. (2010). Characterization of a family with rare deletions in CNTNAP5 and DOCK4 suggests novel risk loci for autism and dyslexia. Biol. Psychiatry.

[40] Bertelsen B., Melchior L., Jensen L.R., Growth C., Glenthøj B., Rizzo R., Debes N.M., Skov L., Brøndum-Nielsen K., Paschou P. (2014). Intragenic deletions affecting two alternative transcripts of the IMMP2L gene in patients with Tourette syndrome. Eur. J. Hum. Genet..

[41] Zhang Y., Liu Y., Zarrei M., Tong W., Dong R., Wang Y., Zhang H., Yang X., MacDonald J.R., Uddin M. (2018). Association of IMMP2L deletions with autism spectrum disorder: A trio family study and meta-analysis. Am. J. Med. Genet. B Neuropsychiatr. Genet..

[42] Gimelli S., Capra V., Di Rocco M., Leoni M., Mirabelli-Badenier M., Schiaffino M.C., Fiorio P., Cuoco C., Gimelli G., Tassano E. (2014). Interstitial 7q31.1 copy number variations disrupting IMMP2L gene are associated with a wide spectrum of neurodevelopmental disorders. Mol. Cytogenet..

[43] Jang W., Kim Y., Han E., Park J., Chae H., Kwon A., Choi H., Kim J., Son J.O., Lee S.J. (2019). Chromosomal Microarray Analysis as a First-Tier Clinical Diagnostic Test in Patients with Developmental Delay/Intellectual Disability, Autism Spectrum Disorders, and Multiple Congenital Anomalies: A Prospective Multicenter Study in Korea. Ann. Lab. Med..

[44] Elia J., Gai X., Xie H.M., Perin J.C., Geiger E., Glessner J.T., D′arcy M., Deberardinis R., Frackelton E., Kim C. (2010). Rare structural variants found in attention-deficit hyperactivity disorder are preferentially associated with neurodevelopmental genes. Mol. Psychiatry.

[45] Viñas-Jornet M., Esteba-Castillo S., Baena N., Ribas-Vidal N., Ruiz A., Torrents-Rodas D., Gabau E., Vilella E., Martorell L., Armengol L. (2018). High Incidence of Copy Number Variants in Adults with Intellectual Disability and Co-morbid Psychiatric Disorders. Behav. Genet..

[46] Qaiser F., Yin Y., Mervis C.B., Morris C.A., Klein-Tasman B.P., Tam E., Osborne L.R., Yuen R.K. (2021). Rare and low frequency genomic variants impacting neuronal functions modify the Dup7q11.23 phenotype. Orphanet J. Rare Dis..

[47] Clarke R.A., Furlong T.M., Eapen V. (2020). Tourette Syndrome Risk Genes Regulate Mitochondrial Dynamics, Structure, and Function. Front. Psychiatry.

[48] Boghosian-Sell L., Comings D.E., Overhauser J. (1996). Tourette syndrome in a pedigree with a 7;18 translocation: Identification of a YAC spanning the translocation breakpoint at 18q22.3. Am. J. Hum. Genet..

[49] Petek E., Windpassinger C., Vincent J.B., Cheung J., Boright A.P., Scherer S.W., Kroisel P.M., Wagner K. (2001). Disruption of a novel gene (IMMP2L) by a breakpoint in 7q31 associated with Tourette syndrome. Am. J. Hum. Genet..

[50] Patel C., Cooper-Charles L., McMullan D.J., Walker J.M., Davison V., Morton J. (2011). Translocation breakpoint at 7q31 associated with tics: Further evidence for IMMP2L as a candidate gene for Tourette syndrome. Eur. J. Hum. Genet..

[51] Rossignol D.A., Frye R.E. (2012). Mitochondrial dysfunction in autism spectrum disorders: A systematic review and meta-analysis. Mol. Psychiatry.

[52] Rose S., Niyazov D.M., Rossignol D.A., Goldenthal M., Kahler S.G., Frye R.E. (2018). Clinical and Molecular Characteristics of Mitochondrial Dysfunction in Autism Spectrum Disorder. Mol. Diagn. Ther..

[53] Fang Z.M., Eapen V., Clarke R.A. (2017). CTNNA3 discordant regulation of nested LRRTM3, implications for autism spectrum disorder and Tourette syndrome. Meta Gene.

[54] Kreilaus F., Chesworth R., Eapen V., Clarke R., Karl T. (2019). First behavioural assessment of a novel Immp2l knockdown mouse model with relevance for Gilles de la Tourette syndrome and Autism spectrum disorder. Behav. Brain Res..

[55] Leung B.K., Merlin S., Walker A.K., Lawther A.J., Paxinos G., Eapen V., Clarke R., Balleine B.W., Furlong T.M. (2023). Immp2l knockdown in male mice increases stimulus-driven instrumental behaviour but does not alter goal-directed learning or neuron density in cortico-striatal circuits in a model of Tourette syndrome and autism spectrum disorder. Behav. Brain Res..

[56] George S.K., Jiao Y., Bishop C.E., Lu B. (2012). Oxidative stress is involved in age-dependent spermatogenic damage of Immp2l mutant mice. Free Radic. Biol. Med..

[57] Ma Y., Mehta S.L., Lu B., Li P.A. (2011). Deficiency in the inner mitochondrial membrane peptidase 2-like (Immp21) gene increases ischemic brain damage and impairs mitochondrial function. Neurobiol. Dis..

[58] Ma Y., Zhang Z., Chen Z., Ma N., Sun S., Zhang J., Ni X., Zhang J., Li P.A. (2017). Suppression of Inner Mitochondrial Membrane Peptidase 2-Like (IMMP2L) Gene Exacerbates Hypoxia-Induced Neural Death Under High Glucose Condition. Neurochem. Res..

[59] He Q., Gu L., Lin Q., Ma Y., Liu C., Pei X., Li P.A., Yang Y. (2020). The Immp2l Mutation Causes Ovarian Aging Through ROS-Wnt/β-Catenin-Estrogen Pathway: Preventive Effect of Melatonin. Endocrinology.

[60] George S.K., Jiao Y., Bishop C.E., Lu B. (2011). Mitochondrial peptidase IMMP2L mutation causes early onset of age-associated disorders and impairs adult stem cell self-renewal. Aging Cell.

[61] Soler R., Füllhase C., Lu B., Bishop C.E., Andersson K.E. (2010). Bladder dysfunction in a new mutant mouse model with increased superoxide—Lack of nitric oxide?. J. Urol..

[62] Han C., Zhao Q., Lu B. (2013). The role of nitric oxide signaling in food intake; insights from the inner mitochondrial membrane peptidase 2 mutant mice. Redox. Biol..

[63] Guimarães-Souza N.K., Yamaleyeva L.M., Lu B., Ramos A.C.M.D.S., Bishop C.E., Andersson K.E. (2015). Superoxide overproduction and kidney fibrosis: A new animal model. Einstein.

[64] Ma Y., Liang R.M., Ma N., Mi X.J., Cheng Z.Y., Zhang Z.J., Lu B.S., Li P.A. (2023). Immp2l Mutation Induces Mitochondrial Membrane Depolarization and Complex III Activity Suppression after Middle Cerebral Artery Occlusion in Mice. Curr. Med. Sci..

[65] Cheng Z., Mi X., Zhang Z., Ma Y. (2021). IMMP2L gene mutation activates mitochondrial apoptotic pathway to aggravate cerebral ischemic injury in mice. Xi Bao Yu Fen Zi Mian Yi Xue Za Zhi.

[66] Liu C., Gu J., Ma W., Zhang Q., Song M., Ha L., Xu X., Jiao H., Huo Z. (2020). Lycium barbarum polysaccharide protects against ethanol-induced spermiotoxicity and testicular degeneration in Immp2l(+/−) mice. Andrologia.

[67] Liu C.L., Zhang Q., Zhang S.-H., Mu C.-L., Yao P., Jiao H.-Y., Xu X., Huo Z.-H. (2021). Lycium barbarum polysaccharide reduces testicular spermatogenic injury in Immp2l−/−mice through GPX4 and AIF pathways. Zhonghua Nan Ke Xue.

[68] Bharadwaj M.S., Zhou Y., Molina A.J., Criswell T., Lu B. (2014). Examination of bioenergetic function in the inner mitochondrial membrane peptidase 2-like (Immp2l) mutant mice. Redox. Biol..

[69] Sun F. (2020). Commentary on “The Immp2l Mutation Causes Ovarian Aging Through ROS-Wnt/beta-Catenin-Estrogen Pathway: Preventive Effect of Melatonin”. Endocrinology.

[70] Wang Z., Xie Y., Chen H., Yao J., Lv L., Li Y., Deng C., Zhang M., Sun X., Liu G. (2021). Guilingji Protects Against Spermatogenesis Dysfunction from Oxidative Stress via Regulation of MAPK and Apoptotic Signaling Pathways in Immp2l Mutant Mice. Front. Pharmacol..

[71] Escalier D. (2008). Knockout mice in the service of reproduction. Gynecol. Obstet. Fertil..

[72] Luo W., Fang H., Green N. (2006). Substrate specificity of inner membrane peptidase in yeast mitochondria. Mol. Genet. Genom..

[73] Moy S.S., Riddick N.V., Nikolova V.D., Teng B.L., Agster K.L., Nonneman R.J., Young N.B., Baker L.K., Nadler J.J., Bodfish J.W. (2014). Repetitive behavior profile and supersensitivity to amphetamine in the C58/J mouse model of autism. Behav. Brain Res..

[74] Berridge K.C., Aldridge J.W., Houchard K.R., Zhuang X. (2005). Sequential super-stereotypy of an instinctive fixed action pattern in hyper-dopaminergic mutant mice: A model of obsessive compulsive disorder and Tourette’s. BMC Biol..

[75] Kalueff A.V., Stewart A.M., Song C., Berridge K.C., Graybiel A.M., Fentress J.C. (2016). Neurobiology of rodent self-grooming and its value for translational neuroscience. Nat. Rev. Neurosci..

[76] Schmeisser M.J., Ey E., Wegener S., Bockmann J., Stempel A.V., Kuebler A., Janssen A.L., Udvardi P.T., Shiban E., Spilker C. (2012). Autistic-like behaviours and hyperactivity in mice lacking ProSAP1/Shank2. Nature.

[77] Kalueff A.V., Aldridge J.W., LaPorte J.L., Murphy D.L., Tuohimaa P. (2007). Analyzing grooming microstructure in neurobehavioral experiments. Nat. Protoc..

[78] Bentley N.L., Fiveash C.E., Osborne B., Quek L.E., Ogura M., Inagaki N., Cooney G.J., Polly P., Montgomery M.K., Turner N. (2018). Protein hypoacylation induced by Sirt5 overexpression has minimal metabolic effect in mice. Biochem. Biophys. Res. Commun..

[79] Montgomery M.K., Osborne B., Brandon A.E., O′Reilly L., Fiveash C.E., Brown S.H., Wilkins B.P., Samsudeen A., Yu J., Devanapalli B. (2019). Regulation of mitochondrial metabolism in murine skeletal muscle by the medium-chain fatty acid receptor Gpr84. FASEB J..

[80] Montgomery M.K., Osborne B., Brown S.H.J., Small L., Mitchell T.W., Cooney G.J., Turner N. (2013). Contrasting metabolic effects of medium- versus long-chain fatty acids in skeletal muscle. J. Lipid Res..

[81] Cohen Hyams T., Mam K., Killingsworth M.C. (2020). Scanning electron microscopy as a new tool for diagnostic pathology and cell biology. Micron.

[82] Mirdita M., Schütze K., Moriwaki Y., Heo L., Ovchinnikov S., Steinegger M. (2022). ColabFold: Making protein folding accessible to all. Nat. Methods.

[83] Pettersen E.F., Goddard T.D., Huang C.C., Meng E.C., Couch G.S., Croll T.I., Morris J.H., Ferrin T.E. (2021). UCSF ChimeraX: Structure visualization for researchers, educators, and developers. Protein Sci..

[84] Elfmann C., Stulke J. (2023). PAE viewer: A webserver for the interactive visualization of the predicted aligned error for multimer structure predictions and crosslinks. Nucleic Acids Res..

[85] Nunnari J., Fox T.D., Walter P. (1993). A mitochondrial protease with two catalytic subunits of nonoverlapping specificities. Science.

[86] Escobar A.P., Martínez-Pinto J., Silva-Olivares F., Sotomayor-Zárate R., Moya P.R. (2021). Altered Grooming Syntax and Amphetamine-Induced Dopamine Release in EAAT3 Overexpressing Mice. Front. Cell. Neurosci..

[87] Ng-Cordell E., Wardell V., Stewardson C., Kerns C.M. (2022). Anxiety and Trauma-Related Disorders in Children on the Autism Spectrum. Curr. Psychiatry Rep..

[88] Perry W., Minassian A., Lopez B., Maron L., Lincoln A. (2007). Sensorimotor Gating Deficits in Adults with Autism. Biol. Psychiatry.

[89] Banker S.M., Gu X., Schiller D., Foss-Feig J.H. (2021). Hippocampal contributions to social and cognitive deficits in autism spectrum disorder. Trends Neurosci..

[90] Gakh O., Cavadini P., Isaya G. (2002). Mitochondrial processing peptidases. Biochim. Biophys. Acta.

[91] Schneider A., Oppliger W., Jeno P. (1994). Purified inner membrane protease I of yeast mitochondria is a heterodimer. J. Biol. Chem..

[92] Burri L., Strahm Y., Hawkins C.J., Gentle I.E., Puryer M.A., Verhagen A., Callus B., Vaux D., Lithgow T. (2005). Mature DIABLO/Smac is produced by the IMP protease complex on the mitochondrial inner membrane. Mol. Biol. Cell.

[93] Shalom D.B. (2009). The medial prefrontal cortex and integration in autism. Neuroscientist.

[94] Rolls E.T., Loh M., Deco G., Winterer G. (2008). Computational models of schizophrenia and dopamine modulation in the prefrontal cortex. Nat. Rev. Neurosci..

[95] Chang W.L., Breier M.R., Yang A., Swerdlow N.R. (2011). Disparate effects of pramipexole on locomotor activity and sensorimotor gating in Sprague-Dawley rats. Pharmacol. Biochem. Behav..

[96] Chen A.P.F., Chen L., Kim T.A., Xiong Q. (2021). Integrating the Roles of Midbrain Dopamine Circuits in Behavior and Neuropsychiatric Disease. Biomedicines.

[97] Swerdlow N.R., Geyer M.A., Braff D.L. (2001). Neural circuit regulation of prepulse inhibition of startle in the rat: Current knowledge and future challenges. Psychopharmacology.

[98] Staton D.M., Solomon P.R. (1984). Microinjections of d-amphetamine into the nucleus accumbens and caudate-putamen differentially affect stereotypy and locomotion in the rat. Physiol. Psychol..

[99] Shafiq S., Pringsheim T. (2018). Using antipsychotics for behavioral problems in children. Expert Opin. Pharmacother..

[100] Zegers-Delgado J., Blanlot C., Calderon F., Yarur H.E., Novoa J., Vega-Quiroga I., Bastias C.P., Gysling K. (2022). Reactive oxygen species modulate locomotor activity and dopamine extracellular levels induced by amphetamine in rats. Behav. Brain Res..

[101] Liang S., Wang X.-L., Zou M.-Y., Wang H., Zhou X., Sun C.-H., Xia W., Wu L.-J., Fujisawa T.X., Tomoda A. (2014). Family-based association study of ZNF533, DOCK4 and IMMP2L gene polymorphisms linked to autism in a northeastern Chinese Han population. J. Zhejiang Univ. Sci. B.

[102] Bjerregaard V.A., Schönewolf-Greulich B., Juel Rasmussen L., Desler C., Tümer Z. (2020). Mitochondrial Function in Gilles de la Tourette Syndrome Patients with and Without Intragenic IMMP2L Deletions. Front. Neurol..

