# Supplementary material for: Antioxidant Behavioural Phenotype in the Immp2l Gene Knock-Out Mouse
Source: Genes (Basel). 2023 Aug 28;14(9):1717. doi: 10.3390/genes14091717 (PMC10531238; doi:10.3390/genes14091717)
Supplement: Supplementary file 1 [file genes-14-01717-s001.zip › genes-2537968-supplementary.pdf]

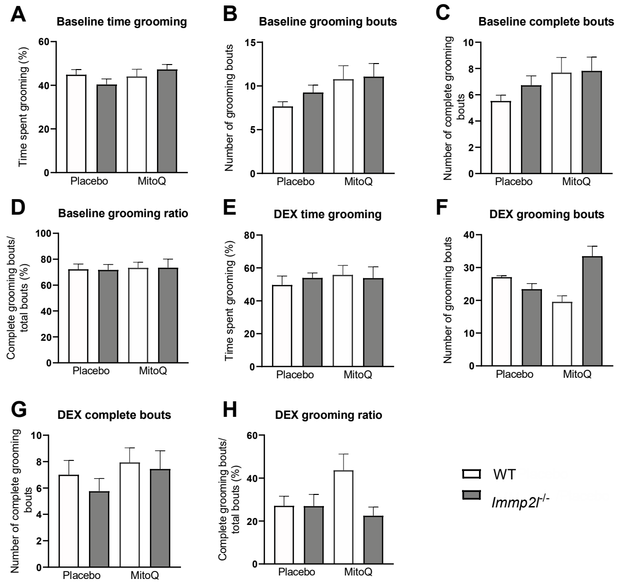

Figure S1. Grooming behaviour in *Immp2l*^{KD} -/- KO mice treated with MitoQ. A-D. Mean time spent grooming, number of grooming bouts, number of completed grooming bouts with the appropriate nose-to-flank sequence, and percentage of completed grooming bouts for *Immp2l*^{KD} -/- KO and wild-type mice treated with MitoQ or placebo (n = 12-15/group). **E-H.** Mean time spent grooming, number of grooming bouts, number of completed grooming bouts with the appropriate nose-to-flank sequence, and percentage of completed grooming bouts for *Immp2l*^{KD} -/- KO and wild-type mice treated with MitoQ or placebo in response to dexamphetamine (n = 8/group). Error bars represent SEM.
